# Validation of a Longitudinal Marker as a Surrogate Using Mediation Analysis and Joint Modeling: Evolution of the PSA as a Surrogate of the Disease‐Free Survival

**DOI:** 10.1002/bimj.70064

**Published:** 2025-06-27

**Authors:** Quentin Le Coent, Catherine Legrand, James J. Dignam, Virginie Rondeau

**Affiliations:** ^1^ ISBA/LIDAM UCLouvain Louvain‐la‐Neuve Belgium; ^2^ Department of Biostatistics Bordeaux Population Health Research Center INSERM U1219 Université de Bordeaux Bordeaux France; ^3^ Department of Public Health Sciences University of Chicago Chicago Illinois USA

**Keywords:** biomarkers, joint modeling, mediation analysis, surrogacy

## Abstract

Longitudinal biomarkers constitute a broad class of potential surrogate endpoints in clinical trials. Several approaches have been proposed for surrogate validation but available methods for validating a longitudinal biomarker as a surrogate of a time‐to‐event endpoint such as death remain limited. In this work, we propose a method for validating a longitudinal outcome as a surrogate of a time‐to‐event endpoint using a combination of joint modeling and mediation analysis. The proportion of the total treatment effect on the time‐to‐event endpoint due to its effect on the biomarker is used as a surrogacy measure. This method is developed to integrate meta‐analytic data using a joint model with random effects at both the individual and trial levels. From this model, the indirect treatment effect through the surrogate as well as the direct and total treatment effects is derived using a mediation formula. A simulation study was designed to evaluate the performance of this approach. We applied this method to a multicentric study on prostate cancer to investigate the use of prostate‐specific antigen level as a surrogate for disease‐free survival.

## 1 Introduction

The use of surrogate endpoints in clinical trials offers important advantages as it can reduce both the duration and the cost of the trial compared to standard clinical endpoints such as overall survival (OS) or disease‐free survival (DFS). Longitudinal biomarkers are an important class of potential surrogates as they directly reflect the evolution of a disease over time. For example, tumor size or prostate‐specific antigen (PSA) levels may indicate how a disease evolves and thus if the treatment is effective. However, when evaluating a treatment, efficacy on clinically relevant endpoints such as OS or DFS will always be the ultimate objective. Therefore, in order for the conclusions of a trial solely based on the surrogate to remain valid regarding the ultimate endpoint, the surrogate must have been previously statistically validated.

Several methods have been proposed in this regard, following the introduction of the Prentice criteria (Prentice 1989) that validate a surrogate if the treatment effect on the final endpoint is null when conditioned on the surrogate. Soon after, Freedman et al. (1992) proposed the proportion of the treatment effect (PTE) on the final endpoint explained by the surrogate as a measure of surrogacy. Since then the methods developed for surrogate validation can be categorized into two main approaches: (1) methods validating surrogates through associations; and (2) methods based on identifications of the similarity of effects between the surrogate and the final endpoint (Joffe and Greene 2009).

For the first approach, the most commonly used method is the meta‐analytic approach that assesses the surrogacy by examining the association between the surrogate and the final endpoint at two levels (Buyse et al. 2000, Daniels and Hughes 1997), considering first the association between the surrogate and the final endpoint at the individual level and then the association between the treatment effects on the surrogate and the final endpoint at the trial level. A putative surrogate will be validated if both associations are deemed as sufficiently strong. Different statistical models were proposed to quantify these associations based on properties of the surrogate and the final endpoint. Renard et al. (2003) extended the two‐stage approach of Buyse et al. (2000) for validating a longitudinal biomarker as a surrogate of a time‐to‐event endpoint. In their work, a joint model was defined and both outcomes were linked together through shared random effects. These random effects were then used to derive the association between both outcomes at the individual and trial levels.

The second approach puts its focus on identification of effects rather than associations: a surrogate will be validated if the PTE on the final endpoint that goes through its effect on the surrogate is sufficiently large. Developments of this second approach have been made using mediation analysis. Frangakis and Rubin (2002) were the first one to use the causal inference framework to study a potential surrogate using principal stratification to define causal effects within subgroups of subjects having the same surrogate outcomes under the two treatment arms. Since then, causal approaches have been proposed by several authors (Conlon et al. 2014, Vandenberghe et al. 2018, Zigler and Belin 2012). However, a limitation of these approaches is that they often use single‐trial data. Moreover, the methods developed for estimating the PTE explained by a surrogate are often limited to cross‐sectional biomarkers (Freedman et al. 1992, Parast et al. 2017, Wang et al. 2020).

In addition, joint models have been proposed, outside the context of surrogate marker validation, to analyze the relationship between a biomarker process and a time‐to‐event outcome (Rizopoulos 2012b, Tsiatis and Davidian 2004, Wulfsohn and Tsiatis 1997). Liu et al. (2018) used such a joint model to examine the causal mechanism in the joint analysis of a longitudinal biomarker and survival endpoint and studied specifically the role played by the random effects regarding the causal identifiability assumptions. In this article, we propose a joint model for the validation of a potential surrogate marker using mediation analysis where the potential surrogate is a longitudinal biomarker and the final endpoint is a time‐to‐event endpoint. Le Coënt et al. (2023) used such a joint modeling approach to derive the PTE explained when both the surrogate and the final endpoint are right‐censored time‐to‐event endpoints in a mediation analysis framework.

Recently, Zheng and Liu (2021) proposed an approach to derive the indirect effect of the treatment on the final endpoint through the surrogate using a joint model combined with mediation analysis. In this approach, the link between both outcomes is made by adjusting for the current level of the biomarker in the hazard function of the final endpoint. This link can be used to derive the effect of biomarker on the final endpoint and then the indirect treatment effect on the final endpoint through the surrogate biomarker. However, this approach does not consider the validation of a potential surrogate based on meta‐analysis (i.e., at the trial level), as the proposed approach was developed for the analysis of a single clinical trial.

Therefore, in this article, we extend the approach of Zheng and Liu (2021) and define a new joint model for a longitudinal biomarker and a time‐to‐event endpoint that also takes into account clustered data from a meta‐analysis (or centers in a multicenter clinical trial). Our model includes random effects both at the individual level and at the trial level. Individual‐level random effects represent individual deviation from the population in terms of intercept and slope in the longitudinal submodel while trial‐level random effects are used to account for the heterogeneity of the treatment effects on both outcomes across trials (or centers). The association between the biomarker process and the final outcome is modeled through a link function in the survival submodel. The choice of the link function depends on the underlying biomarker process; examples of potential links that we will discuss in more detail include the current level or the current slope of the biomarker process.

From this model, a decomposition of the total treatment effect (TTE) on the final endpoint as an indirect effect through the biomarker and a direct effect can be derived. The (time‐dependent) proportion of the indirect over the total effect of the treatment on the final endpoint can then be used as a measure of surrogacy. Our approach allows researchers to estimate these quantities while acknowledging the meta‐analytic nature of the data.

This article is organized as follows. In Section 2, we introduce the proposed joint model and define the related direct and indirect treatment effect as well as the proposed surrogacy measure. In Section 3, we present a simulation study to investigate the performances of this method regarding the estimation of the parameters of the model as well as the measure of surrogacy. In Section 4, we apply this method to a multicenter study on prostate cancer to evaluate the PSA evolution over time as a surrogate for DFS. Finally, in Section 5, we conclude and provide some comments and further developments on the proposed methodology.

## 2 Methods

### 2.1 Notations, Potential and Observed Outcomes

Let K be the number of trials (or centers) in the study and $K_{i}$ the number of patients in the ith trial. In the following, we denote by ij the jth patient from the ith trial. Let $Z_{ij}$ be the binary treatment assignment, where $Z_{ij}=1$ denotes the experimental arm and $Z_{ij}=0$ the control arm and let $X_{ij}$ be a vector of covariates. We now introduce the counterfactual and observed outcomes for the biomarker and the final outcome, respectively.

Let $M_{ij}^{z}\left(t\right)$ be the potential counterfactual biomaarker process at time t had subject ij been assigned to treatment z. The whole potential biomarker path is denoted by $M_{ij}^{z}≔\left\{M_{ij}^{z}\left(t\right),0\leq t\leq \tau \right\}$ where τ is the maximum follow‐up time. The biomarker path for subject ij is $M_{ij}^{Z_{ij}}=\left\{M_{ij}^{Z_{ij}}\left(t_{ijk}\right),k=1,\cdots ,n_{ij}\right\}$ where $n_{ij}$ is the number of repeated observations and $t_{ijn_{ij}}\leq \tau$ are the timepoints at which the biomarker is observed. Moreover, this biomarker might be measured with errors and we denote by ${\tilde{M}}_{ij}^{Z_{ij}}$ the observed with errors repeated biomarker measurements of subject ij. To ease the notation in the following, we use $M_{ij}$ and ${\tilde{M}}_{ij}$ for $M_{ij}^{Z_{ij}}$ and ${\tilde{M}}_{ij}^{Z_{ij}}$, respectively. In the following, we denote by $\bar{M}\left(t\right)$ the trajectory of M up to time t: $\bar{M}\left(t\right)=\left\{M\left(u\right),0\leq u\leq t\right\}$. Let $T_{ij}^{zm}$ be the counterfactual survival time of subject ij if the treatment is fixed to z and the biomarker path to m. Here, m denotes an infinite‐dimensional trajectory over time. The survival and censoring time of subject ij are then $T_{ij}^{Z_{ij}M_{ij}^{Z_{ij}}}$ and $C_{ij}=C_{ij}^{Z_{ij}M_{ij}^{Z_{ij}}}$, respectively, which correspond to the potential outcomes of subject ij given the treatment $Z_{ij}$ and biomarker $M_{ij}$. As for the biomarker, to facilitate the notation in the following, we will use $T_{ij}$ and $C_{ij}$ instead of $T_{ij}^{Z_{ij}M_{ij}^{Z_{ij}}}$ and $C_{ij}=C_{ij}^{Z_{ij}M_{ij}^{Z_{ij}}}$, respectively. Given the survival context, the observed survival outcomes are $T_{ij}^{*}=\min\left(T_{ij},C_{ij}\right)$ and $\delta_{ij}=I\left(T_{ij}\leq C_{ij}\right)$. Finally, for subject ij the vector of observed data is $O_{ij}=\left({\tilde{M}}_{ij},T_{ij}^{*},\delta_{ij},Z_{ij},X_{ij}\right)$.

### 2.2 Mediation Analysis

Given the survival context, we define the following survival function:

$$S^{zz^{\prime }}\left(t\right)=P\left(T_{ij}^{zM^{z^{\prime }}}\geq t\right),\;\;0\leq t\leq \tau .$$

The function $S^{zz^{\prime }}\left(t\right)$ is the survival function of the potential outcome 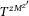 where the treatment value is set to z for the survival outcome but to $z^{\prime }$ for the biomarker. From $S^{zz^{\prime }}\left(t\right)$, the TTE on the survival scale can be expressed as

$$\mathrm{TTE}\left(t\right)=S^{11}\left(t\right)-S^{00}\left(t\right),$$

which is the difference of survival when the treatment is set to 1 for both T and M (i.e., the experimental arm) versus when the treatment is set to 0 (the control arm). This TTE can further be decomposed into a natural indirect effect (NIE) and a natural direct effect (NDE):

$$\begin{array}{cc} \mathrm{TTE}(t) & =S^{11}\left(t\right)-S^{00}\left(t\right)\\ & =\underset{\mathrm{NIE}(t)}{\underset{︸}{S^{11}\left(t\right)-S^{10}\left(t\right)}}+\underset{\mathrm{NDE}(t)}{\underset{︸}{S^{10}\left(t\right)-S^{00}\left(t\right)}}. \end{array}$$

The NIE is the difference of survival when the treatment is set to 1 for both outcomes versus when the treatment is set to 1 only for the survival outcome. Therefore, the only difference between these survivals is the treatment status for the biomarker and hence it quantifies the treatment effect on the survival due to its effect on the biomarker. In an analogous way, the NDE quantifies the treatment effect on the survival independent of the biomarker. From this decomposition of the TTE into a direct and indirect effect, the PTE can be used as a measure of surrogacy, defined as the ratio of indirect effect over total effect:

$$\mathrm{PTE}\left(t\right)≔\frac{\mathrm{NIE}(t)}{\mathrm{TTE}(t)}.$$

A surrogate will be validated if the value PTE(t) is large enough. While there is no acknowledged threshold in the literature, a value of PTE(t) larger than 0.8 (i.e., more than 80% of the treatment effect on the final endpoint is due to the biomarker) is typically considered sufficiently large for a potential surrogate to be validated. Moreover, PTE(t) is a function of time, but for practical purposes it could be more relevant to only consider specific and clinically relevant time‐points to validate a surrogate.

We now introduce a joint model adapted for the context of meta‐analyses and show how we can derive an expression to compute the functions $S^{zz^{\prime }}\left(t\right)$ from the observed data based on this model.

### 2.3 Joint Model

As announced in Section 2.1, let $M_{ij}\left(t\right)$ denote the true value of the biomarker for subject ij at time t and ${\tilde{M}}_{ij}\left(t\right)$ be the observed biomarker. The joint model for $M_{ij}^{z}$ and $T_{ij}^{zM^{z^{\prime }}}$ is

(1)

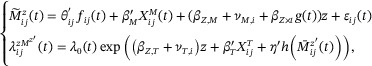

where $M_{ij}^{z}\left(t\right)=\theta_{ij}^{\prime }f\left(t\right)+\beta_{M}^{\prime }X_{ij}^{M}\left(t\right)+\left(\beta_{Z,M}+\nu_{M,i}+\beta_{Z\times t}g\left(t\right)\right)z$ is the true, error‐free, biomarker value and $\varepsilon_{ij}\left(t\right)∼N\left(0,\sigma_{\varepsilon }^{2}\right)$ are the independent measurement errors.

The vector $f_{ij}\left(t\right)$ represents the temporal evolution of the biomarker and may be composed of several components. The temporal evolution of the biomarker is not assumed to have any particular shape and can be more or less flexible depending on the setting and the biomarker. The associated vector $\theta_{ij}=\beta +\omega_{ij}$ is the sum of fixed effects (β) and individual random effects ($\omega_{ij}$) associated with each component of $f_{ij}\left(t\right)$. For example, if $f_{ij}\left(t\right)={\left(1,t\right)}^{\prime }$, then $\theta_{ij}={\left(\beta_{0}+\omega_{ij0},\beta_{1}+\omega_{ij1}\right)}^{\prime }$ which gives a random intercept and random slope model. The function g(t) is used to take into account a potential interaction between time and treatment. The vector ω is assumed to be Gaussian with mean 0 and covariance matrix $\Sigma_{\omega }$. The function $\lambda_{0}\left(t\right)$ is the baseline hazard function that is estimated in a parametric but flexible way using cubic M‐splines (Joly et al. 1998). The parameters $\beta_{Z,M}$ and $\beta_{Z,T}$ are the fixed treatment effects on the biomarker and the time‐to‐event, respectively. The random effects $\left(\nu_{M,i},\nu_{T,i}\right)$ are trial‐level effects taking into account the heterogeneity of the treatment effects on both outcomes across trials and are assumed to be jointly Gaussian:

$${\left(\nu_{M,i},\nu_{T,i}\right)}^{\prime }∼N\left(\mu_{\nu },\Sigma_{\nu }\right),\;\mu_{\nu }=\left(\begin{array}{c} 0\\ 0 \end{array}\right),\;\Sigma_{\nu }=\left(\begin{array}{cc} \sigma_{\nu_{M}}^{2} & \sigma_{\nu_{M,T}}\\ \sigma_{\nu_{M,T}} & \sigma_{\nu_{T}}^{2} \end{array}\right).$$

**FIGURE 1:**
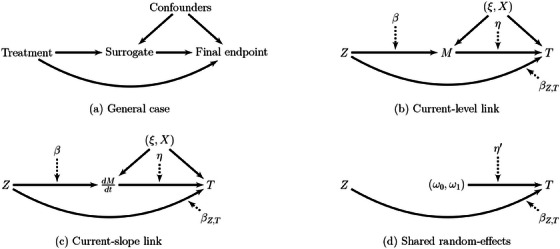
Pathways of mediation of the treatment on the final endpoint T in: the general setting (a), the “current‐level” link function (b), the “current‐slope” link (c), and the “shared random effects” case (d) in the joint model 1. The solid lines represent the link between the outcomes with the associated parameters in the model that represents this link.

Given the circumstances, we will show in the simulations that in some cases these random effects can be either ignored or replaced by fixed effects to simplify the computations. The covariates $X_{ij}^{M}$ and $X_{ij}^{T}$ are included in the biomarker and time‐to‐event submodel, respectively, with their associated vector of coefficients $\beta_{M}$ and $\beta_{T}$. These covariates are not necessarily mutually exclusive and can share the same components. The function h(·) is the link function between the longitudinal marker and the event process. Common choices of link functions include $h\left(\cdot \right)=\left(\omega_{0},\omega_{1}\right)$ (shared random effects), h(·)=M(t) (current value of the biomarker), and $h\left(\cdot \right)=\frac{dM(t)}{dt}$ (current slope). Parameters η are association parameters between the biomarker and the event time process. From a mediation analysis point of view, a mediated effect of the treatment on the final endpoint through the biomarker is possible only if the quantities in the link function depend on the treatment. In the following, we focus mainly on the current‐value and current‐slope link functions. Figure 1b represents the case with a current‐level link function and the associated parameters of model 1 while Figure 1c concerns the case of the current‐slope link in which the arrow between Z and $\frac{dM(t)}{dt}$ only exists if the treatment as an effect on the slope, for example, by including an interaction between time and treatment. The case with shared random effects does not allow a mediated effect since the individual random effects are independent of the treatment (Figure 1d).

Let ϕ being the whole vector of parameters of the model, that is,

$$\phi =(\lambda_{0},\eta ,\sigma_{\varepsilon },\Sigma_{\omega },\Sigma_{\nu },\beta ,\beta_{T},\beta_{M},\beta_{Z,T},\beta_{Z,M}).$$

Estimator of ϕ, $\hat{\phi }$, is obtained by maximizing the penalized likelihood using the Levenberg–Marquardt algorithm (Marquardt 1963, Rondeau et al. 2007),

(2)

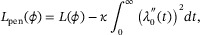

where L(ϕ) is the full likelihood of the data and 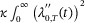 is a penalization term on the second‐order derivative of the baseline hazard function resulting in a smooth estimate. The larger κ is, the smoother the estimated function ${\hat{\lambda }}_{0}\left(t\right)$ will be. This parameter can be chosen using cross‐validation. Details on the likelihood construction and computation are provided in Web Appendix A. Standard errors of the parameters of the model can be estimated through the inverse of Hessian matrix of the penalized likelihood evaluated at $\hat{\phi }$. In the following, we note by ξ the vector of the random effects in the model (both individual‐ and trial‐level random effects). One of the difficulty in the maximization of the (log) likelihood comes from the integration over the random effects. We have chosen a Monte‐Carlo approach for integrating over the trial‐level random effects and a pseudo‐adaptive Gauss–Hermite rule for integrating over the individual‐level random effects (Rizopoulos 2012a).

### 2.4 Derivation of Mediation Quantities

In order to derive quantities related to mediation analysis and the potential outcomes distributions, we must rely on some identifiability assumptions (Imai et al. 2010).

First, the stable unit treatment value assumption (SUTVA) requires that each version of the treatment is well‐defined and that there is no interference between an individual treatment assignment and another's outcome. The first part of the SUTVA is reasonable for randomized clinical trials in which each version of the treatment is defined without ambiguity. The second part of the SUTVA may not hold in particular settings, even in randomized clinical trials. For example, in infectious diseases, if a patient is in contact with another whose risk of getting infected depends on the treatment, then the potential outcome of this patient not only depends on the treatment he/she receives but also on the treatment the other patient receives. However, for other settings, such as oncology in our case the second part of the SUTVA is almost certain to be met.

We also require the consistency assumption which states that, for subject ij, the biomarker process $M_{ij}$ equals the potential process associated with the treatment actually assigned to this patient, $M_{ij}=M_{ij}^{Z_{ij}}$. In the same way, the event time $T_{ij}$ equals $T_{ij}^{Z_{ij}M_{ij}}$ as well as the censoring time $C_{ij}$ is $C_{ij}^{Z_{ij}M_{ij}}$. We want to point out that generally, the consistency assumption requires that the biomarker process M is measured without error. However, this is likely not to be the case and in a joint modeling perspective where we are interested by determining the true biomarker process which is the observed process without the error term. Hence, we do not require the “no measurement‐error” assumption but rather assume that the longitudinal model for the true process is correctly specified.

We also make an adapted version of the sequential ignorability assumption (Imai et al. 2010, Le Coënt et al. 2023):

- $\left(T_{ij}^{z^{\prime }m},M_{ij}^{z},C_{ij}^{zm}\right)⊥\;\;\;\;⊥Z_{ij}|\left(X_{ij},\xi_{ij}\right)$, $\forall z,z^{\prime },m$,
- $\left(T_{ij}^{z^{\prime }m},C_{ij}^{z^{\prime }m}\right)⊥\;\;\;\;⊥M_{ij}^{z}|\left(Z_{ij}=z,X_{ij},\xi_{ij}\right)$, $\forall z,z^{\prime },m$.

This version of the sequential ignorability assumption states that all the confounding between the biomarker and the time‐to‐event is captured by either the covariates and/or the random effects. The use of random effects thus makes this assumption more flexible as they can capture the confounding not taken into account by the covariates. Recall from Section 2.2 that we are interested in computing $S^{zz^{\prime }}\left(t\right)$, the survival function of T when the treatment Z is set to the value z for T but when the biomarker M behaves as if Z was set to $z^{\prime }$. Using the SUTVA and the consistency assumption we have, for covariates x (Tchetgen 2011),

(3) $$S^{zz^{\prime }}\left(t|x\right)=\int_{\xi }S_{T}\left(t|h({\bar{M}}^{z^{\prime }}\left(t\right)),\xi ,Z=z,X=x\right)f_{\xi }\left(\xi \right)d\xi ,$$

where $S_{T}\left(t|h({\bar{M}}^{z^{\prime }}\left(t\right)),\xi ,Z=z,X=x\right)$ is the survival function of T conditional on $h({\bar{M}}^{z^{\prime }}\left(t\right))$, the treatment Z, covariates X, and random effects ξ:

$$\begin{array}{cc} & S_{T}\left(t|h({\bar{M}}^{z^{\prime }}\left(t\right)),\xi ,Z=z,X=x\right)\\ & \;=\exp\left[-\int_{0}^{t}\lambda_{0}\left(u\right)\exp\left(\left(\beta_{Z,T}+\nu_{T}\right)z+\beta_{T}^{\prime }X^{T}\left(u\right)+h({\bar{M}}^{z^{\prime }}\left(t\right)\right)du\right]. \end{array}$$

The function $f_{\xi }\left(\xi \right)$ in Formula (3) is the joint density of the random effects (individual and trial levels). The integral over these random effects ξ can be carried out using either a Monte‐Carlo method or Gaussian quadrature.

It should be noted that according to model (1), the quantity involved in h(·) only depends on the covariates and the random effects. Hence, as opposed to Zheng and Liu (2021) who assume that the error term ε is included in the argument of the link function h, we do not need to integrate over the distribution of ε once we have conditioned on z, x, and ξ.

From $S^{zz^{\prime }}\left(t|x\right)$, we can derive the direct and indirect effect of the treatment for a given level of covariates x as

$$\begin{array}{cc} & \mathrm{NIE}\left(t|x\right)=S^{11}\left(t|x\right)-S^{10}\left(t|x\right),\\ & \mathrm{NDE}\left(t|x\right)=S^{10}\left(t|x\right)-S^{00}\left(t|x\right). \end{array}$$

In order to obtain these effects for the overall population, we can integrate over the covariates

$$\begin{array}{cc} & \mathrm{NIE}\left(t\right)=\int_{X}\left(S^{11}\left(t|x\right)-S^{10}\left(t|x\right)\right)f_{X}\left(x\right)dx,\\ & \mathrm{NDE}\left(t\right)=\int_{X}\left(S^{10}\left(t|x\right)-S^{00}\left(t|x\right)\right)f_{X}\left(x\right)dx. \end{array}$$

These integrals can be approximated using Monte Carlo by considering that the observed covariates ${\left(X_{i}\right)}_{1\leq i\leq n}$ in the data set is a random sampling from $f_{X}\left(x\right)$ and then computing NIE(t∣x) and NDE(t∣x) for each $X_{i}$ in the data set and averaging them all. In the case where the covariates are categorical we only need to compute the NIE and NDE for each category and then take their sum weighted by the proportion of each category in the data. From the estimated parameters $\hat{\phi }$ of the model, we can thus derive estimates of the indirect and direct treatment effects, $\hat{\mathrm{NIE}}\left(t\right)$ and $\hat{\mathrm{NDE}}\left(t\right)$, respectively, and compute an estimation of PTE(t):

$$\hat{\mathrm{PTE}}\left(t\right)=\frac{\hat{\mathrm{NIE}}\left(t\right)}{\hat{\mathrm{NIE}}\left(t\right)+\hat{\mathrm{NDE}}\left(t\right)}.$$

Estimated standard errors and confidence bands for the function $\hat{\mathrm{PTE}}\left(t\right)$ can be obtained using parametric bootstrap based on the asymptotic Gaussian distribution of the maximum likelihood estimator $\hat{\phi }$. By sampling ϕ from this distribution and computing PTE(t) for each draw, we can obtain percentile‐based confidence bands, for example, by using the 2.5th and 97.5th percentiles of the obtained PTE(t) at each time t.

## 3 Simulations

### 3.1 Scenarios and Data Generation

We ran a simulation study to evaluate the finite sample performances of our proposed approach.

We considered the three following cases. In the first case, no treatment heterogeneity was introduced. In the second case, a small heterogeneity of the treatment effects was simulated with a large number trials (100). This scenario aims to mimic a multicentric study where many centers are present but where a small treatment heterogeneity across the centers can be expected since they all follow the same protocol. The third case considered a larger heterogeneity and a fewer number of trials (10). This scenario aims at reproducing the conditions of a meta‐analysis of multiple clinical trials where the number of available trials remains relatively low and one can expect a larger heterogeneity of the treatment effects across the trials. To reduce the computation time of the simulations, we did not include trial‐level random effects in the models fitted to these simulated data. Instead, for the first and second scenarios, the heterogeneity of the treatment effects was not taken into account. For the third scenario, it was taken into account using fixed effects for the 10 trials in both the survival and longitudinal submodels, which required the estimation of 18 additional parameters. For each scenario, the number of subjects in the data set was fixed and set to 800. Each patient was randomly assigned trial j (j=1,⋯,K) with probability $K^{-1}$. The binary treatment Z was randomly generated with $P\left(Z=1\right)=P\left(Z=0\right)=\frac{1}{2}$. The assumed simulated model is given by

(4)

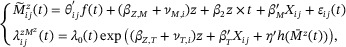

where

$$f\left(t\right)=\left(\begin{array}{c} 1\\ t \end{array}\right),\;\theta_{ij}=\left(\begin{array}{c} \beta_{0}+\omega_{ij0}\\ \beta_{1}+\omega_{ij1} \end{array}\right).$$

Parameters $\beta_{0},\beta_{1}$, and $\beta_{2}$, are, respectively, the fixed intercept, slope, and the effect of the treatment on the slope. The trial‐level random effects $\left(\nu_{M,i},\nu_{T,i}\right)$ were generated from a centered Gaussian distribution with covariance matrix

$$\Sigma_{\nu }=\left(\begin{array}{cc} \sigma_{\nu_{M}}^{2} & \sigma_{\nu_{M,T}}\\ \sigma_{\nu_{M,T}} & \sigma_{\nu_{T}}^{2} \end{array}\right).$$

For the second scenario, the covariance matrix $\Sigma_{\nu }$ used to generate the trial‐specific treatment effects $\nu_{M}$ and $\nu_{T}$ is given by, $\Sigma_{\nu }=\left(\begin{array}{cc} 0.086 & 0.056\\ 0.056 & 0.086 \end{array}\right)$ while for the third scenario the covariance matrix used is, $\Sigma_{\nu }=\left(\begin{array}{cc} 0.400 & 0.280\\ 0.280 & 0.400 \end{array}\right)$. The individual‐level random effects $\omega_{ij}$ were also generated from a bivariate‐centered Gaussian distribution with covariance matrix,

$$\left(\begin{array}{c} \omega_{ij0}\\ \omega_{ij1} \end{array}\right)∼N\left(0,\Sigma_{\omega }\right),\;\Sigma_{\omega }=\left(\begin{array}{cc} \sigma_{\omega_{0}}^{2} & \sigma_{\omega_{01}}\\ \sigma_{\omega_{01}} & \sigma_{\omega_{1}}^{2} \end{array}\right).$$

The covariate $X_{ij}$ was binary variable such that $P\left(X_{ij}=0\right)=P\left(X_{ij}=1\right)=0.5$. The link function $h(M^{Z})$ used was the current‐level link:

$$\begin{array}{cc} h({\bar{M}}_{ij}^{z}\left(t\right)) & =E(M_{ij}^{z}\left(t\right)|\omega_{ij})\\ & =\beta_{0}+\omega_{ij0}+\left(\beta_{1}+\omega_{ij1}\right)t+\beta_{Z,M}z+\beta_{2}z\times t+\beta_{M}^{\prime }X_{ij}. \end{array}$$

Follow‐up times and censoring indicators were simulated in order to yield approximately 20% of censoring. The average number of repeated measurements per subject was ≈6.2. Regarding the surrogacy measures, we focused on three timepoints at t=1,2, and 3 corresponding to approximately 50%, 75%, and 80% of survival. The true values for PTE(·), NIE(·), and NDE(·) displayed in the results (Tables 1, 2, and 3) were computed by using the values of the parameters used for the simulations in the formula in Equation (3) to derive $S^{zz^{\prime }}\left(t|x\right)$ and then the corresponding values of PTE(t), NIE(t), and NDE(t).

**TABLE 1:** Simulations results for the scenario without trial‐level heterogeneity.

	True	Est.^a^	SE.^b^	SD.^c^	CR.^d^
*Survival part*					
Association	0.300	0.299	0.026	0.043	0.740
Covariate (X)	−0.300	−0.298	0.074	0.078	0.945
Treatment	1.000	1.023	0.078	0.092	0.885
*Longitudinal part*					
Intercept ($\beta_{0}$)	0.200	0.188	0.026	0.093	0.570
Slope ($\beta_{1}$)	1.200	1.307	0.011	0.215	0.165
Covariate (X)	0.100	0.102	0.025	0.105	0.585
Treatment	1.000	0.999	0.036	0.096	0.705
Treatment‐time interaction	0.300	0.242	0.034	0.212	0.385
*Random effects*					
Residuals	0.300	0.277	0.005	0.017	0.310
$\sigma_{\omega_{0}}^{2}$	0.562	0.558	0.030	0.037	0.905
$\sigma_{\omega_{1}}^{2}$	0.450	0.339	0.024	0.153	0.250
$\sigma_{\omega_{01}}$	−0.112	−0.080	0.020	0.043	0.515
*Mediation measures*					
PTE(1)	0.280	0.271	0.037	0.043	0.917
PTE(2)	0.177	0.167	0.024	0.040	0.769
PTE(3)	0.099	0.088	0.016	0.032	0.620
NIE(1)	−0.122	−0.118	0.018	0.020	0.934
NIE(2)	−0.067	−0.064	0.007	0.017	0.661
NIE(3)	−0.020	−0.019	0.003	0.008	0.496
NDE(1)	−0.313	−0.317	0.022	0.026	0.893
NDE(2)	−0.309	−0.319	0.024	0.030	0.860
NDE(3)	−0.186	−0.189	0.028	0.030	0.909

^a^ Mean of the estimated parameters.

^b^ Mean of the estimated standard errors.

^c^ Empirical standard deviation.

^d^ Coverage rate of the 95% confidence interval.

**TABLE 2:** Simulations results for the scenario with 100 centers and a small heterogeneity of the treatment effects across trials

	True	Est.^a^	SE.^b^	SD.^c^	CR.^d^
*Survival part*					
Association	0.300	0.317	0.026	0.041	0.745
Covariate (X)	−0.300	−0.290	0.074	0.079	0.910
Treatment	1.000	0.951	0.078	0.094	0.850
*Longitudinal part*					
Intercept ($\beta_{0}$)	0.200	0.195	0.025	0.118	0.535
Slope ($\beta_{1}$)	1.200	1.329	0.011	0.218	0.160
Covariate (X)	0.100	0.109	0.025	0.117	0.560
Treatment	1.000	0.998	0.036	0.103	0.585
Treatment‐time interaction	0.300	0.217	0.031	0.243	0.365
*Random effects*					
Residuals	0.300	0.277	0.005	0.017	0.295
$\sigma_{\omega_{0}}^{2}$	0.562	0.603	0.033	0.050	0.770
$\sigma_{\omega_{1}}^{2}$	0.450	0.351	0.025	0.163	0.275
$\sigma_{\omega_{01}}$	−0.112	−0.082	0.022	0.041	0.590
*Mediation measures*					
PTE(1)	0.280	0.294	0.038	0.050	0.870
PTE(2)	0.177	0.190	0.027	0.054	0.770
PTE(3)	0.099	0.108	0.022	0.056	0.780
NIE(1)	−0.122	−0.124	0.018	0.024	0.880
NIE(2)	−0.067	−0.071	0.007	0.021	0.600
NIE(3)	−0.020	−0.023	0.003	0.011	0.480
NDE(1)	−0.313	−0.295	0.022	0.029	0.800
NDE(2)	−0.309	−0.296	0.024	0.034	0.840
NDE(3)	−0.186	−0.179	0.027	0.034	0.870

^a^ Mean of the estimated parameters.

^b^ Mean of the estimated standard errors.

^c^ Empirical standard deviation.

^d^ Coverage rate of the 95% confidence interval.

**TABLE 3:** Simulations results for the scenario with 10 trials and a large heterogeneity of the treatment effects across trials

	True	Est.^a^	SE.^b^	SD.^c^	CR.^d^
*Survival part*					
Association	0.300	0.334	0.029	0.052	0.689
Covariate (X)	−0.300	−0.288	0.077	0.080	0.932
Treatment	1.000	0.986	0.084	0.221	0.558
*Longitudinal part*					
Intercept ($\beta_{0}$)	0.200	0.130	0.046	0.275	0.321
Slope ($\beta_{1}$)	1.200	1.256	0.012	0.223	0.153
Covariate (X)	0.100	0.109	0.025	0.090	0.495
Treatment	1.000	1.045	0.037	0.233	0.237
Treatment‐time interaction	0.300	0.110	0.057	0.359	0.221
*Random effects*					
Residuals	0.300	0.280	0.005	0.015	0.300
$\sigma_{\omega_{0}}^{2}$	0.562	0.681	0.037	0.097	0.316
$\sigma_{\omega_{1}}^{2}$	0.450	0.381	0.027	0.254	0.242
$\sigma_{\omega_{01}}$	−0.112	−0.085	0.023	0.051	0.526
*Mediation measures*					
PTE(1)	0.280	0.297	0.086	0.093	0.844
PTE(2)	0.177	0.205	0.086	0.107	0.642
PTE(3)	0.099	0.137	1.176	0.102	0.624
NIE(1)	−0.122	−0.119	0.041	0.040	0.917
NIE(2)	−0.067	−0.083	0.022	0.050	0.587
NIE(3)	−0.020	−0.042	0.016	0.040	0.606
NDE(1)	−0.313	−0.290	0.054	0.080	0.761
NDE(2)	−0.309	−0.305	0.042	0.067	0.798
NDE(3)	−0.186	−0.221	0.053	0.079	0.716

^a^ Mean of the estimated parameters.

^b^ Mean of the estimated standard errors.

^c^ Empirical standard deviation.

^d^ Coverage rate of the 95% confidence interval.

### 3.2 Simulations Results

The parameters estimation of the proposed model, direct effect, indirect effect, and PTE(t) were investigated in terms of bias, mean estimated standard errors, empirical standard deviation, and coverage rate of the 95% confidence intervals. Since NDE(t), NIE(t), and PTE(t) are functions of time we chose three timepoints at which they were evaluated.

Table 1 shows the results of the simulations in which no heterogeneity for the treatment effects on the survival and the longitudinal outcomes were introduced. Hence, in this scenario, the model estimated was correctly specified regarding the simulated data. From this table, we can see that the model parameters are well estimated, except for those related to the slope in the longitudinal submodel ($\beta_{1}$ for the fixed effect which is overestimated and $\sigma_{\omega_{1}}^{2}$ for the variance of the random slope for which the standard deviation is underestimated). Estimations regarding mediation measures are also correct. For PTE(t), we see that the difference between the estimated standard errors and the standard deviation is increased for larger values of t which yields lower coverage rate of the 95% confidence intervals.

Table 2 shows the result for the first scenario based on data generated with a small treatment by center heterogeneity and a large number of centers (100). In this scenario, this heterogeneity was not taken into account to fit our model. Results from Table 2 show that in such a context, ignoring the heterogeneity over centers still leads to correct estimated values for both the model parameters and mediation measures while dramatically reducing the computation time. In this table, we see that the treatment effect estimate is only slightly biased in the survival submodel (mean estimated value of 0.951 compared to the true value of 1) and correctly estimated in the longitudinal submodel. The coverage rate for the treatment effect in the longitudinal submodel (0.585) is under the nominal value of 0.950 but good for the survival part. Regarding the mediation measures, the bias observed in the treatment effect on the survival outcome also results in a small bias for PTE(t), NIE(t), and NDE(t). However, this bias seems more limited for NIE(t) for which the coverage rates of the 95% confidence intervals remain acceptable.

Table 3 shows the simulations results for the scenario in which the number of trial was 10 and with a larger heterogeneity of the treatment effects. In this scenario, the heterogeneity of the treatment effects was taken into account by using fixed effects for the trials. Therefore, 18 (9 per submodel) additional parameters were estimated (estimations not shown in the table). From Table 3, we see that the parameters are well estimated but the treatment effect in the longitudinal part is slightly biased upward (mean estimated value of 1.045 to be compared with the true value of 1). This results in a small coverage rate of the 95% confidence interval. However, the mean estimate (0.986 compared to the true value 1) of the treatment effect in the survival submodel is better. For the mediation measures, we can see that there is some bias for estimation of PTE(t). This bias is smaller for smaller values of t. For all the results in the three tables, we also see that for both NIE(t) and NDE(t), the estimations of both the values and the standard errors are better for smaller values of t. This can be explained by the lower number of observed events in the data as t increases. This suggests that these quantities should not be computed for value of too large values of t.

In addition, PTE(t) should not be computed for small values of t for which the survival rates between the experimental and control groups are too close to show a significant treatment effect. The PTE(t) being defined as the ratio of indirect effect over total effect, the total effect is likely to be close to 0 for small values of t, which will make difficult to derive a meaningful interpretation for PTE(t). Therefore, the surrogacy measures NIE(t), NDE(t), and PTE(t) should be only computed for values of t that are not too close to the baseline or the end of the study.

The standard errors of the parameters in the longitudinal submodel, especially those related to the temporal evolution of the biomarker, underestimate the standard deviation which is a common issue in joint models for longitudinal and survival data.

When heterogeneity is introduced in the data (scenarios of Tables 2 and 3) we see that the variances of the random intercept, slope, and residuals are biased. This suggests that introducing an heterogeneity not taken into account affects the estimation of these quantities.

These results from Tables 1, 2, and 3 also suggest that not including the trial‐level random effects in the model (which increase the computation time) does not have a large influence on the estimation of PTE when the trial‐level heterogeneity is low. This is the case for example in multicentric studies in which the number of centers can be large and the heterogeneity of the treatment effects across centers can be small due to all centers following the same protocol. However, for real meta‐analysis where the number of trials is usually small (often around 10–15) but with potentially a more important heterogeneity of the treatment effects between the trials it should be preferable to use a model in which this heterogeneity is taken into account. The results of Table 3 suggest that the use of fixed effects can be sufficient to take this heterogeneity into account without having to fit a more computationally intense model with trial‐level random effects.

## 4 Application

We applied the proposed approach to a multicenter study on locally advanced prostate cancer (Horwitz et al. 2008). We investigated the evolution of PSA over time as a potential surrogate of the DFS. In this application, the centers (institutions) were used as clustering units for the meta‐analytic approach.

Previous approaches for validating the PSA as a surrogate were undertaken by Armstrong et al. (2007) where they investigated the PSA as a surrogate of the OS in the context of metastatic prostate cancer. The authors used the PTE on the OS going through the treatment effect on a PSA decline ≥30% at 3 months. They concluded that this proportion is approximately two‐thirds. However, their analysis is limited by the use of a single‐time value (decline/nondecline) and the PTE is estimated from fitting two separate Cox models on time‐to‐death (one using only the treatment arm and the other adjusting on both the treatment arm and the surrogate). The PTE is taken as one minus the ratio of coefficient of the treatment effects in the second (adjusted model) versus that in unadjusted model. Since the surrogate is a posttreatment variable likely to be influenced by the treatment, raw adjustment on the surrogate creates a selection bias by not taking into account potential confounders between the surrogate and the final endpoint which may result in a lack of causal interpretability. Moreover, taking estimated regression coefficients as measures of “effects” renders difficult the interpretation of the PTE on other scale, such as the survival scale.

### 4.1 Data Description

The trial evaluated the benefit of adding a long‐term androgen deprivation therapy (LTAD) for patients with T2c–T4 prostate cancer undergoing radiotherapy. Patients were randomized to either radiotherapy with a short‐term androgen deprivation therapy (STAD arm) or to radiotherapy with an additional 24 months of ADT (LTAD arm). For this analysis, the definitive clinical endpoint is the DFS defined as the time to occurrence of a local progression, distant metastasis, a biochemical failure, or death, whichever occurred first. Biochemical failure was defined as either three consecutive PSA rises after a posttreatment nadir, receiving additional ADT or a PSA greater than 4 ng/mL, whichever occurred first (Horwitz et al. 2008). For practical purposes, we focused on an analysis censored at 5 years, although trial follow‐up continued beyond this point for long‐term DFS and other endpoints.

Of 1423 patients, 703 were randomized to the STAD arm and the remaining 716 patients were included in the LTAD arm. This study was a multicentric trial with 117 centers that we used as the basis for our meta‐analytic approach. The number of observed events was 495 in the LTAD arm and 563 in the SDAT arm. Table 4 provides a description of the patient's characteristics and follow‐up in each treatment arm. Previous results showed that at 5 years, the LTAD arm was superior to STAD regarding the DFS (46.4% vs. 28.1%, p<0.001) (Hanks et al. 2003, Horwitz et al. 2008). Kaplan–Meier estimates of the DFS for each treatment arm are available in Figure 1 of the Web Appendix C.

**TABLE 4:** Patient's characteristics (based on a 5‐year follow‐up).

Variable	LTAD (N=716)	STAD (N=703)
Age	69.82 (43, 88)	69.58 (43, 87)
iPSA^a^	2.98 (−0.69, 5.21)	3.04 (−1.50, 5.52)
Gleason		
<7	291 (41%)	290 (41%)
7	251 (35 %)	226 (32%)
>7	174 (24 %)	187 (27%)
Clinical stage		
T2	331 (46%)	323 (46%)
T3–T4	385 (54%)	380 (54%)
Number of PSA measures	10.03 (1, 18)	8.50 (1, 18)
Mean time between two measures^b^	0.37 (0, 2.89)	0.38 (0, 3.51)
Median follow‐up	5.0 (0.0, 5.0)	3.27 (0.0, 5.0)
DFS events	328 (46%)	432 (61%)
Cause of DFS events		
Death	79 (24%)	66 (15%)
Local progression	60 (18%)	87 (20%)
Distant metastasis	13 (4%)	12 (3%)
Biochemical failure	176 (54%)	267 (62%)

*Note:* Continuous variables: Mean (Minimum, Maximum);

Categorical variables: Count (Percentage).

^a^ PSA at study entry (in the log(·+0.1) scale).

^b^ Time expressed in years.

### 4.2 Data Analysis and Results

**FIGURE 2:**
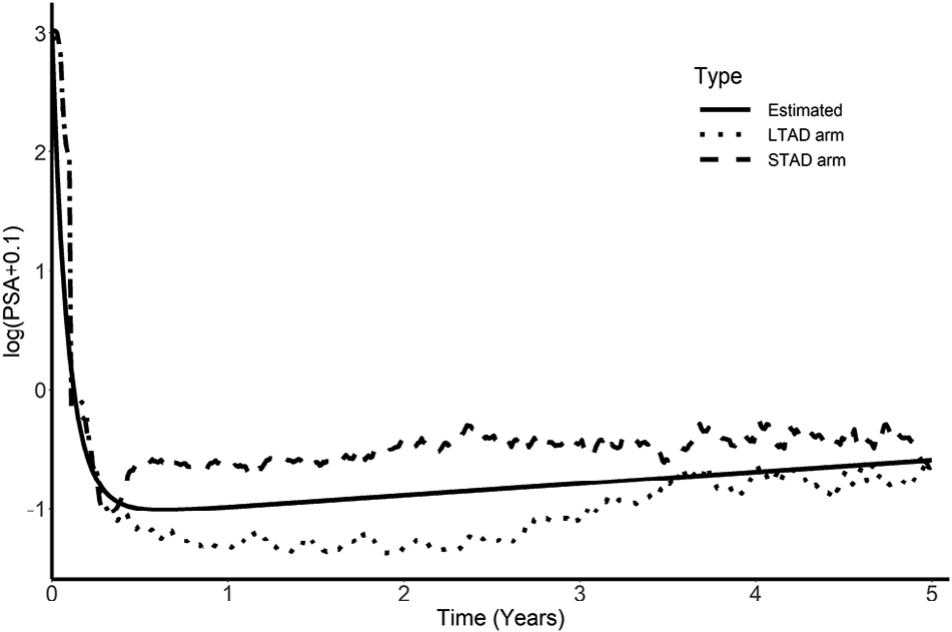
Average PSA trajectory (in log(·+0.1) scale) in both treatment arms (dotted and dashed lines) and the estimated curve based on a mixed model (solid line).

According to previous longitudinal analyses of PSA, we used a log transformation of the PSA in the longitudinal submodel instead of the raw value of PSA (Ferrer et al. 2016, Proust‐Lima and Taylor 2009, Sène et al. 2014, Taylor et al. 2013). This is mainly due to the “exponential” increase of the PSA. Figure 2 shows the average (across patients) trajectory of the log‐transformed PSA over time for the STAD arm (dashed curve) and the LTAD arm (dotted curve). The shape indicates a sharp decrease in PSA after baseline (due to patients being treated with radiotherapy) and an almost linear increase of the PSA during the following years. From this figure, we can see that the differences in trajectories between both groups may mainly lies in different levels of PSA rather than in different slopes, suggesting that the “current‐level” association may be more relevant from a surrogacy point of view than the slope (since the treatment may have no effect on the slope).

To take into account the specific evolution of the log PSA over time, we included two functions of time to modelize both the sharp decrease of the PSA following baseline, $f_{1}\left(t\right)$, and the linear increase in the long term, $f_{2}\left(t\right)$. According to previous analyses (Ferrer et al. 2016, Proust‐Lima and Taylor 2009), we used functions of the following form: $f_{1}\left(t\right)={\left(1+t\right)}^{\alpha }-1$ and $f_{2}\left(t\right)=\frac{t^{\lambda +1}}{{\left(1+t\right)}^{\lambda }}$. The parameters α and λ were estimated using profile likelihood on mixed models on the PSA evolution through a grid search which yielded α=−11.4 and λ=−0.3. The resulting estimated curve is shown in Figure 2 (solid curve). Prior analysis on a reduced model based only on the longitudinal PSA suggested that of the three main components of the PSA trajectories (intercept, $f_{1}\left(t\right)$, and $f_{2}\left(t\right)$), there was an individual heterogeneity only on the intercept and $f_{2}\left(t\right)$. As a result, in the joint model, we only included individual random effect for these two components.

We used the following joint model:

(5)

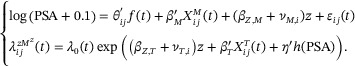

The covariates $X_{ij}^{M}$ and $X_{ij}^{T}$ included are the Gleason score and the clinical stage. Here, we have $f\left(t\right)={\left(1,f_{1}\left(t\right),f_{2}\left(t\right)\right)}^{\prime }$. We estimated two models: one with a “current‐level” link function and another with a “current‐slope” link. In the latter case, from a mediation viewpoint, in order to enable a potential mediated effect the slope must be dependent on the treatment Z. Therefore, we added an interaction term (in both models) between the treatment and the long‐term increase of the PSA represented by $f_{2}\left(t\right)$. Hence, $\theta_{ij}^{\prime }f\left(t\right)$ is as follows:

$$\theta_{ij}^{\prime }f\left(t\right)={\left(\begin{array}{c} \beta_{0}+\omega_{ij0}\\ \beta_{1}\\ \beta_{2}+\omega_{ij2}+\beta_{3}Z_{ij} \end{array}\right)}^{\prime }\times \left(\begin{array}{c} 1\\ f_{1}\left(t\right)\\ f_{2}\left(t\right) \end{array}\right),$$

with ${\left(\omega_{ij0},\omega_{ij2}\right)}^{\prime }∼N\left(0,\Sigma_{\omega }\right)$ the individual‐level random effects. The two link functions used are


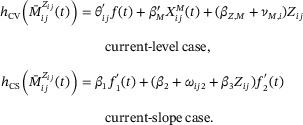

**FIGURE 3:**
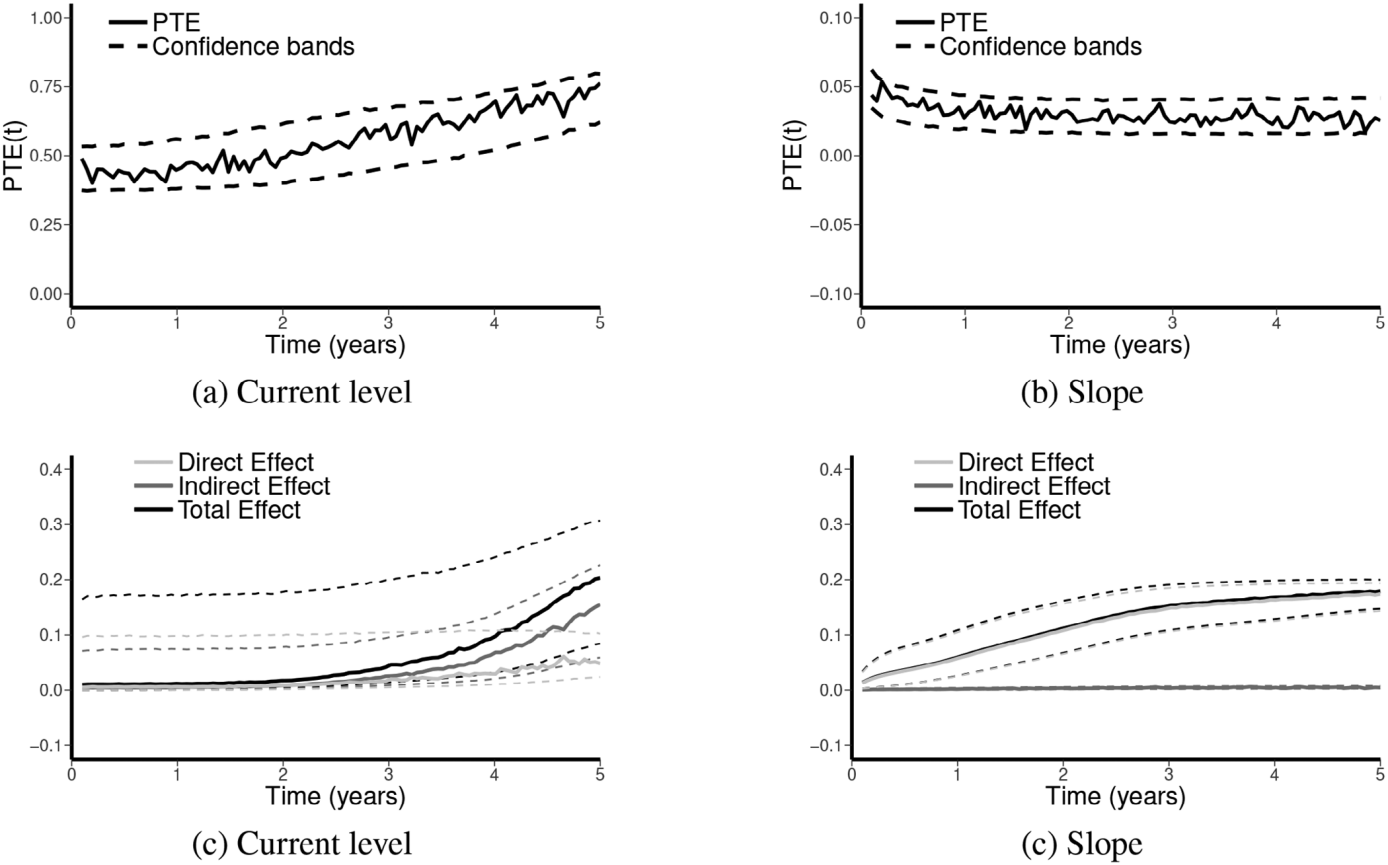
Results from the application on prostate cancer. Top panel is the estimated PTE for the “current‐level” (left) and “current‐slope” links. Bottom panel displays the associated estimated natural direct, indirect, and total effects. Dashed curves are the 95% pointwise confidence bands. Note that for readability the scales between panels (a) and (b) are different.

Estimated results are shown in Table 5. For the evaluation of PTE(t), we selected three timepoints, respectively, at 1, 3, and 5 years. From Table 5, we see that in both the current‐level and current‐slope cases there is a significant treatment effect on both the longitudinal evolution of the PSA and the final endpoint. The treatment significantly reduces the average level of PSA ($\beta_{Z,M}$) but also its long‐term increase (parameter $\beta_{3}$). The estimated variances of trial‐level random effects ($\sigma_{M}$ and $\sigma_{T}$) suggest that there is no heterogeneity of the treatment effects on both endpoints across centers. This is not surprising since there centers were all part of the same clinical trial and thus likely followed all the same treatment procedures and follow‐ups, smoothing out any heterogeneity of the treatment effects. For both the current‐level and current‐slope link, the association (η) between the longitudinal evolution of PSA over time and the DFS process is significant. Therefore, a significant effect of the treatment on PSA and a significant effect of the PSA on the DFS (either by its current value or slope) suggests that there is a mediated effect. However, the magnitude of this mediated effect has to be compared to the direct treatment effect on the event time process (parameter $\beta_{Z,T}$). Moreover, even if the association is significant in both cases, the association in the current‐value case is much stronger than in the current‐slope case (η=1.336 vs. 0.106). Therefore, the mediated effect is more important for the current‐level link which can be seen in the estimated PTE. For the current‐level link, PTE ranges from 0.472 to 0.762 while for the current slope it ranges from 0.024 to 0.034. The evolution of PTE over time is given in the top panel of Figure 3, with the PTE for the current level and slopes links on the left and right, respectively. The associated estimated natural direct, indirect, and total effects are represented in the bottom panel. The confidence bands for these functions were obtained using parametric bootstrap with 2000 samplings. These results suggest that the slope (even if significantly associated with the time‐to‐event process) is a poor surrogate as it mediates less than 3% of the TTE on the DFS. On the contrary, the current level seems to be a more interesting surrogate which mediates a much larger PTE, with a trend over time showing an increase from 50% to 75%. This increase over time might be explained by the significant interaction between the treatment and the long‐term increase of the PSA, $f_{2}\left(t\right)$. Since $f_{2}\left(t\right)$ is increasing, this interaction have more weight over time which might explain the increasing PTE over time seen in Figure 3. These results suggest that the current level of PSA might be an interesting surrogate of the DFS, especially for long‐term benefit. This analysis on real data set has probably no bias estimation since it has the advantage to consider also trial‐level random effects in order to take into account the heterogeneity of the treatment effects on both outcomes across trials.

**TABLE 5:** Results of the application on prostate cancer.

	Current level				Current slope		
	Est.	Std. err.	p‐Value		Est.	Std. err.	p‐Value
*Mediation measures*							
PTE(1)	0.472	0.038			0.034	0.008	
PTE(3)	0.559	0.056			0.024	0.005	
PTE(5)	0.762	0.045			0.026	0.007	
NIE(1)	0.005	0.018			0.002	0.001	
NIE(3)	0.026	0.023			0.004	0.001	
NIE(5)	0.155	0.041			0.005	0.001	
NDE(1)	0.006	0.029			0.062	0.018	
NDE(3)	0.020	0.027			0.149	0.019	
NDE(5)	0.048	0.019			0.174	0.013	
*Longitudinal submodel*							
Intercept ($\beta_{0}$)	3.282	0.060	<0.001		3.313	0.060	<0.001
$f_{1}\left(t\right)$ ($\beta_{1}$)	4.277	0.021	<0.001		4.269	0.021	<0.001
$f_{2}\left(t\right)$ ($\beta_{2}$)	0.590	0.029	<0.001		0.481	0.030	<0.001
Treatment ($\beta_{Z,M}$)	−0.257	0.035	<0.001		−0.297	0.037	<0.001
Treatment × $f_{2}\left(t\right)$ ($\beta_{3}$)	−0.192	0.018	<0.001		−0.141	0.018	<0.001
Gleason^a^							
=7	0.055	0.037	0.137		0.064	0.038	0.088
<7	0.162	0.040	<0.001		0.168	0.041	<0.001
Clinical stage^b^							
T3–T4	0.126	0.032	<0.001		0.122	0.033	<0.001
*Survival submodel*							
Association (η)	1.336	0.064	<0.001		0.106	0.004	<0.001
Treatment ($\beta_{Z,T}$)	−0.360	0.100	<0.001		−0.495	0.077	<0.001
Gleason^a^							
=7	0.117	0.094	0.214		0.087	0.088	0.325
>7	0.166	0.098	0.090		0.354	0.091	<0.001
Clinical stage^b^							
T3–T4	0.083	0.080	0.307		0.190	0.075	0.010
*Random effects*							
*Individual level*							
$\sigma_{\omega_{0}}$	0.300	0.017			0.310	0.018	
$\sigma_{\omega_{2}}$	0.079	0.004			0.069	0.004	
$\sigma_{\omega_{02}}$	−0.010	0.006			−0.011	0.006	
*Trial level*							
$\sigma_{\nu_{M}}$	0.084	0.021			0.147	0.013	
$\sigma_{\nu_{T}}$	0.008	0.003			0.005	0.003	
$\sigma_{\nu_{M,T}}$	−0.015	0.002			−0.025	0.002	
Residuals (σ)	0.605	0.004			0.607	0.004	

^a^ Reference group: Gleason <7.

^b^ Reference group: Clinical stage T2.

## 5 Discussion

In this article, we proposed an approach to validate a longitudinal biomarker as a surrogate of a time‐to‐event using mediation analysis and joint modeling. An advantage of the proposed method is that it can be applied to meta‐analytic data while most of the developments for surrogate validation using mediation analysis were restricted to single‐trial data. The use of meta‐analytic data introduces heterogeneity (and especially heterogeneity of the treatment effects) within the validation process and therefore strengthen it.

When studying a longitudinal biomarker as a surrogate of a time‐to‐event endpoint, the trajectory of the biomarker process must be carefully modeled and the nature of the association between the two outcomes should be thoughtfully considered. Especially, one should consider which component of the trajectory is the most relevant from a surrogacy point of view. For example, is a sudden increase or decrease of the biomarker more informative than its value? If so, including the slope of the biomarker in the link function may be more relevant than its current level. In this article, we considered two options: either the value itself or the slope but a combination of the two is also possible. These are single‐time values (the slope or the value at time t) but an aggregate over time may also be relevant. For example, the cumulative biomarker values $\int_{0}^{t}M\left(u\right)du$ have been previously proposed as an association term in joint modeling (Rizopoulos 2012b). Regarding the trajectory of the biomarker over time, we used a simple linear mixed model but other approaches based on mechanistic model have been proposed in the context of joint modeling (Król et al. 2018).

However, we have to be aware that from a mediation analysis perspective, the association term included in the link function should depend on the treatment. A commonly used link function in joint models is the shared random‐effects link where the individual random intercept and slope ($\omega_{0}$ and $\omega_{1}$ in the notations of our model) are included in the survival submodel. These random effects are independent of the treatment, thus no matter the treatment effect on the biomarker, there will be no mediated treatment effect on the event time process through the random effects. Similarly, for mediation to be possible in the case where the slope is included as the association term, the slope should depend on the treatment effect. The magnitude of this effect will yield either a strong or weak mediated effect, but not allowing the association term to be influenced by the treatment will lead, by model definition, to the absence of mediation.

In this article, we mainly focused on deriving the PTE on the final endpoint going through its effect on the biomarker using mediation analysis but as stated in the introduction other approaches have been proposed (Burzykowski et al. 2005). These approaches use meta‐analytic data to derive association between the surrogate and the final endpoint at the individual level as well as at the trial level through the use of random effects. It should be noted that even if we focused on mediation, these two‐level associations can also be computed from our proposed model. The trial‐level association can be derived from the covariance matrix of the trial‐level random effects $\Sigma_{\nu }$ as the coefficient of determination $R_{\text{trial}}^{2}=\frac{\sigma_{\nu_{M,T}}^{2}}{\sigma_{\nu_{M}}^{2}\sigma_{\nu_{T}}^{2}}$. One possibility to look at the individual‐level association is to compute the Kendall's τ between the biomarker M and the event time T. In the case of shared random effects, the Kendall's τ only depends on the distribution of the individual‐level random effects and can be computed (Sofeu et al. 2019). However, when the link function includes either M or its slope the derivation of a formula for the Kendall's τ may be more complicated as this link should be taken into account.

Besides these measures of associations, another measure of surrogacy proposed is the surrogate threshold effect. Its purpose is to predict the treatment effect on the final endpoint from its estimated effect on the surrogate (Burzykowski and Buyse 2006). In the setting of shared‐ or correlated random effects, the surrogate and final endpoint are assumed independent given the random effects. This can be translated as removing the arrow from M to T in Figure 1a. In that case, the TTE on the final endpoint is $\beta_{T}+\nu_{T}$ and one needs only to focus on predicting it from the observed effect of the treatment on the surrogate. However, when there is a direct link between the biomarker and the final endpoint, predicting the total effect of the treatment on the final endpoint requires to also take into account the effect of the biomarker on the final endpoint which renders the computation more complicated.

It should be noted that our main surrogacy measure, the PTE, is mostly dependent on the indirect treatment effect through the surrogate. Therefore, a null indirect treatment effect will rule out a potential surrogate. However, it is not a requirement that a good surrogate must induce a strong indirect effect (VanderWeele 2013). It could be the case that there is no indirect treatment effect on the final endpoint through the surrogate, but because both endpoints are strongly associated together through a common cause, then the surrogate, even if inducing a null indirect treatment effect, could still be a good surrogate. This is illustrated in Figure 2 in the Supporting Information. The upper figure shows the setting of the mediation analysis where the indirect effect is composed of two direct effects: the direct treatment effect on the surrogate and the direct effect of the surrogate on the final endpoint. The surrogate will be rule out from a mediation perspective whenever one of these effects is null. However, the lower figure illustrates a setting in which a surrogate can still be a good one even if there is no indirect treatment effect. In our approach, the common is represented by random effects in the joint model.

While in our approach the PTE is the main measure of surrogacy, its raw value does not carry much information regarding surrogacy without looking also carefully at the direct and indirect effects separately. Indeed, if both effects are in the opposite direction then the total effect might be smaller than the indirect effect and therefore the PTE can take values larger than one. We recommend that the interpretation of the PTE should always be done after looking at the direct and indirect effects separately.

In our application, DFS is a composite endpoint defined as the earliest of either death, biochemical failure, distant metastasis, or local recurrence. In particular, the definition of a biochemical failure is based on the evolution of the PSA, which creates de facto a link between DFS and the biomarker.

It should be assessed if studying surrogacy in cases where the final endpoint definition is partly based on the biomarker is relevant. In our case, it could also be assessed if the use of biochemical failures as composite events has more influence in a model based on the current level or the current slope of the PSA. In the definition of a biochemical failure, both the current value of the PSA (value greater than 4 ng/mL) and slope (three consecutive rises) intervene so that it seems likely that the use of biochemical failures influences both cases.

One possible extension of our approach is to combine the current level and the current slope as surrogates of a survival endpoint. This approach would combine both, using two association parameters $\eta_{1}$ and $\eta_{2}$, but might complicate the computation and the interpretation of the results in terms of surrogacy and mediation.

The estimation procedure in our approach requires numerical approximations in the likelihood computation, especially regarding numerical integrations. The computation of the likelihood involves two integral approximations: one over the distribution of the random effects and one over time when integrating the baseline hazard function to derive the survival function. Improving the accuracy of these integrals will help achieve better estimation of the parameters of the model. The integral over the random effects is currently approximated using the Gauss–Hermite quadrature whose accuracy can be improved by increasing the number of nodes it uses. However, with two individual‐level random effects the number of evaluations of the likelihood required is the square of the number of nodes and therefore, for a large number of nodes computation times can therefore be prohibitive. The integral over time is currently approximated using the Gauss–Kronrod (GK) quadrature, the accuracy of the numerical approximation could also be increased by using more nodes in the procedure. However, in the current implementation of the GK quadrature used, the number of nodes cannot fixed and cannot be modified. Other integration techniques could be used to improve the accuracy of these approximations.

Regarding computation time, it should be noted that including trial‐level random effects in the model can increase the time needed for the model to run. Including these random effects implies that in the evaluation of the likelihood we have to numerically approximate the integral over the distribution of these random effects. For this numerical approximation, several methods can be used, such as Gauss–Hermite quadrature or Monte‐Carlo simulations. From our own experience, the latter is to be preferred when the dimension of the random effects is at least two.

Other approaches have been studied for hierarchical joint models. Sudell et al. (2019) investigated several approaches for clustered joint models such as using fixed cluster‐specific effects, random effects, or stratified baseline hazard functions. However, their approaches mostly on joint model with shared random‐effects link functions. Brilleman et al. (2019) proposed a Bayesian approach for hierarchical joint model. While they primarily focused on models in which patients represent the higher clustering unit (other units are lesions within patients and repeated biomarker measurements within lesions), they discuss extensions to meta‐analytic or multicentric settings.

The use of fixed effects to take into account the trial‐level heterogeneity could be an alternative to the random effects approach that is less time‐consuming. The results of Table 3 show that using fixed effects can give satisfactory results for the estimation of the mediation measures. However, for K trials one has to estimate 2(K−1) additional parameters and therefore for large value of K the estimation of the model can become cumbersome. Regarding the use of fixed effects, our recommendations are as follow. In the case where we expect a small heterogeneity of the treatment effects (especially for multicentric studies), ignoring this heterogeneity can still give appropriate results (see results of Table 2). In the case where there is likely a strong heterogeneity of the treatment effects (e.g., in a meta‐analysis) but a reasonable number of trials then one can use fixed effects for the trials indicator variable (see results of Table 3). In the case where there are many trials/centers and a large heterogeneity of the treatment effects then one will have to rely on the use of trial‐level random effects.

Finally, in this article we focused on validating a single biomarker as a surrogate using a joint model, but joint modeling has been extended in order to take into account the effect of several biomarkers on a time‐to‐event (Rustand et al. 2022). It would be an interesting to extend this approach in the case of several biomarkers.

## 6 Software

The method is implemeted in the longiPenal function of the R package frailtypack (Król et al. 2017) which is available on R‐CRAN at https://CRAN.R‐project.org/package=frailtypack. A working example available in the package can be run using the following call R command to call model (1) is,

data(colorectal)
data(colorectalLongi)
# Retrieve last observation for each corresponding to survival information
colorectalSurv $<‐$ subset(colorectal, new.lesions == 0)
# Modify treatment variables into binary variables
colorectalSurv$treatment<‐sapply(colorectalSurv$treatment,function(t) ifelse(t==“S”,1,0))
colorectalLongi$treatment<‐sapply(colorectalLongi$treatment,function(t) ifelse(t==“S”,1,0))
# Fit the joint model with current‐level link function and mediation analysis
# Program takes around 10 minutes to run
mod.col=longiPenal(Surv(time1, state) age+treatment,
tumor.size age+year*treatment,
data=colorectalSurv, data.Longi = colorectalLongi, random = c(“1”, “year”),
id = “id”, link = “Current‐level”,timevar=“year”,method.GH = “Pseudo‐adaptive”,
mediation = TRUE,med.trt = colorectalSurv$treatment,
med.center = NULL,med.nmc = 1000,n.knots = 7, kappa = 5,n.nodes = 9,
pte.times=c(1,1.5,2),pte.boot = F,pte.nmc = 5000,pte.nboot = 1000)
print(mod.col)




The arguments formula and formula.longi define the covariate used in the survival and longitudinal submodel, respectively. The link argument specifies the type of link function used in the survival submodel and mediation is a Boolean argument specifying if a mediation analysis should be performed. timevar specifies the time variable in data.Longi related to measurements time of the biomarker. The argument med.trt specifies the treatment indicator for each subject. The user can specify the timepoints at which the mediation and surrogacy quantities must be evaluated through the argument pte.times.

After the execution of the program, summary of the estimations and related figures (Kaplan–Meier estimates, baseline hazards, PTE(t), and natural effects) can be obtained from the functions print(), summary(), and plot(). The output of the command print(mod.col) is available in Web Appendix D.

## Conflicts of Interest

The authors declare no conflicts of interest.

## Open Research Badges

This article has earned an Open Data badge for making publicly available the digitally‐shareable data necessary to reproduce the reported results. The data is available in the Supporting Information section.

This article has earned an open data badge “**Reproducible Research**” for making publicly available the code necessary to reproduce the reported results. “The results reported in this article were reproduced partially due to data confidentiality.”

## Supporting information


**Supporting file 1:** bimj70064‐sup‐0001‐SuppMat.pdf.

**Supporting file 2:** bimj70064‐sup‐0002‐Datacode.zip.

## Data Availability

The data that support the findings of this study are available from the corresponding author upon reasonable request.
