# Supplementary material for: Validation of a Longitudinal Marker as a Surrogate Using Mediation Analysis and Joint Modeling: Evolution of the PSA as a Surrogate of the Disease‐Free Survival
Source: Biom J. 2025 Jun 27;67(4):e70064. doi: 10.1002/bimj.70064 (PMC12205229; doi:10.1002/bimj.70064)
Supplement: Supplementary file 1 — Supporting file 1: bimj70064‐sup‐0001‐SuppMat.pdf. [file BIMJ-67-e70064-s002.pdf]

Supporting information for "Validation of a longitudinal marker as a surrogate using mediation analysis and joint modeling: evolution of the PSA as a surrogate of the disease-free survival" by Q. Le Coënt, J. Dignam, C. Legrand and V. Rondeau.

Web Appendix A: Likelihood construction and computation

Likelihood of the model

For individual ij the time-to-event and censored times are denoted T_{ij} and C_{ij}. The observed longitudinal biomarker is $\tilde{M}_{ij} = \{\tilde{M}_{ijk} = \tilde{M}_{ij}(t_{ijk}), 1 \leq k \leq n_{ij}\}$. The follow-up time is $T^*_{ij} = \min(T_{ij}, C_{ij})$ with its associated indicator $\delta_{ij} = \mathbb{1}_{T_{ij} \leq C_{ij}}$. The observed data for patient ij are therefore,

$$\mathcal{O}_{ij} = \left(T^*_{ij}, \delta_{ij}, M_{ij}, Z_{ij}, X_{ij}\right).$$

The joint model is given by,

$$\begin{cases} \tilde{M}^z_{ij}(t) = \theta'_{ij} f(t) + \beta'_M X^M_{ij} + (\beta_{Z,M} + \nu_{M,i}) z + \varepsilon_{ij}(t) \\ \lambda^{zM^{z'}}_{ij}(t) = \lambda_0(t) \exp\left((\beta_{Z,T} + \nu_{T,i})\, z + \beta'_T X^T_{ij} + \eta' h\left(M^{z'}\right)\right), \end{cases} \tag{1}$$

where the random effects $(\nu_{M,i}, \nu_{T,i})$ are trial-level effects taking into account the heterogeneity of the treatment effect across trial and are assumed to be jointly gaussian:

$$(\nu_{M,i}, \nu_{T,i})^\intercal \sim \mathcal{N}\left(\mu_\nu, \Sigma_\nu\right), \quad \mu_\nu = \begin{pmatrix} 0 \\ 0 \end{pmatrix}, \ \Sigma_\nu = \begin{pmatrix} \sigma^2_{\nu_M} & \sigma_{\nu_{TM}} \\ \sigma_{\nu_{TM}} & \sigma^2_{\nu_T} \end{pmatrix}.$$

The function $f(t)$ represents the temporal evolution of the biomarkers and may be composed of several components. The parameter θ is the sum of fixed effects and individual random effects assiocated with each component of $f(t)$, $\theta_{ij} = \beta + \omega_{ij}$. For example if $f(t) = \begin{pmatrix} 1 \\ t \end{pmatrix}$ then $\theta_{ij} = \begin{pmatrix} \beta_0 + \omega_{ij0} \\ \beta_1 + \omega_{ij1} \end{pmatrix}$. The vector ω is assumed to be gaussian with mean 0 and covariance matrix Σ_ω. The error terms $\varepsilon_{ij}(t)$ are assumed independent and normally distributed: $\varepsilon_{ij}(t) \sim \mathcal{N}(0, \sigma_\varepsilon)$. The baseline hazard function $\lambda_0(t)$ is estimated using cubic M-splines. If K knots are used, there are two boundary knots and $k-2$ inner knots, and $k+2$ parameters $(\Xi_1, \ldots, \Xi_{K+2})$. Let G

be the number of trials, $(n_1, \dots, n_G)$ the number of patients in each trials and n_{ij} the number of measurements for patient j from trial i. Let ϕ be the vector of all the parameters of the model

$$\phi = (\lambda_{0,T}, \eta, \sigma_\varepsilon, \Sigma_\omega, \Sigma_\nu, \beta_T, \beta_M, \beta, \beta_{Z,T}, \beta_{Z,M}).$$

The complete likelihood of the data is the product of the contribution of each trial

$$L(\phi) = \prod_{i=1}^{G} L_i(\phi)$$

where $L_i(\phi)$ is the contribution of the ith trial. Let ν_i be the trial-level random effects, $\nu_i = (\nu_{T,i}, \nu_{M,i})$ Given, ν_i the patients from trial i are independent. Hence,

$$\begin{aligned}
L_i(\phi) &= \int_{\mathbb{R}^2} L_i(\phi, \nu_i) \mathrm{d}\nu_i \\
&= \int_{\mathbb{R}^2} L_i(\phi \mid \nu_i) f(\nu_i) \mathrm{d}\nu_i \\
&= \int_{\mathbb{R}^2} \left(\prod_{j=1}^{n_i} L_{ij}(\phi \mid \nu_i) \right) f(\nu_i) \mathrm{d}\nu_i
\end{aligned}$$

where $L_{ij}(\phi \mid \nu_i)$ is the individual contribution of subject j from trial j. We have,

$$\begin{aligned}
L_{ij}(\phi \mid \nu_i) &= p_\phi(T^*_{ij}, \delta_{ij}, \tilde{M}_{ij} \mid \nu_i) \\
&= \int_{\mathbb{R}^2} p_\phi(T^*_{ij}, \delta_{ij}, \tilde{M}_{ij}, \omega_{ij} \mid \nu_i) \mathrm{d}\omega_{ij} \\
&= \int_{\mathbb{R}^2} p_\phi(T^*_{ij}, \delta_{ij}, \tilde{M}_{ij} \mid \nu_i, \omega_{ij}) f(\omega_{ij}) \mathrm{d}\omega_{ij} \\
&= \int_{\mathbb{R}^2} p_\phi(T^*_{ij}, \delta_{ij} \mid \nu_i, \omega_{ij}, \tilde{M}_{ij},) p_\phi\left(\tilde{M}_{ij} \mid \nu_i, \omega_{ij}\right) f(\omega_{ij}) \mathrm{d}\omega_{ij}.
\end{aligned}$$

Assuming that the censoring time C is independent of T given M, X and Z and non informative we have

$$\begin{aligned}
p_\phi\left(T^*_{ij}, \delta_{ij} \mid \nu_i, \omega_{ij}, \tilde{M}_{ij}\right) &= \left(\lambda(T^*_{ij} \mid \nu_i, \omega_{ij}, \tilde{M}_{ij}) S(T^*_{ij} \mid \nu_i, \omega_{ij}, \tilde{M}_{ij})\right)^{\delta_{ij}}) S(T^*_{ij} \mid \nu_i, \omega_{ij}, \tilde{M}_{ij})^{1-\delta_{ij}} \\
&= \lambda(T^*_{ij} \mid \nu_i, \omega_{ij}, \tilde{M}_{ij})^{\delta_{ij}} S(T^*_{ij} \mid \nu_i, \omega_{ij}, \tilde{M}_{ij}) \\
&= \left[\lambda_0(T^*_{ij}) \exp\left((\beta_{Z,T} + \nu_{T,i}) Z_{ij} + \beta_T^\intercal X_{ij}(t) + h\left(M_{ij}\right)\right)\right]^{\delta_{ij}} \times \\
&\quad \exp\left(- \int_0^{T^*_{ij}} \lambda_0(u) \exp\left((\beta_{Z,T} + \nu_{T,i}) Z_{ij} + \beta_T^\intercal X_{ij}(u) + h\left(M^*_{ij}\right)\right) \mathrm{d}u \right)
\end{aligned}$$

where the integral

$$\int_0^{Y_{ij}} \lambda_0(u) \exp\left((\beta_{Z,T} + \nu_{T,i}) Z_{ij} + \beta_T^\intercal X_{ij}(u) + h\left(M_{ij}\right)\right) \mathrm{d}u$$

needs to be computed over the trajectory of $h\left(M_{ij}\right)$. The longitudinal part of the individual contribution given the random effects is

$$p_\phi\left(\tilde{M}_{ij} \mid \nu_i, \omega_{ij}\right) = \frac{1}{(2\pi\sigma^2)^{n_{ij}/2}} \exp\left\{ - \frac{\|\tilde{M}_{ij} - \theta_{ij}^\intercal f(t) + \beta_M^\intercal X_{ij}^M + (\beta_{Z,M} + \nu_{M,i}) Z_{ij}\|^2}{2\sigma^2} \right\}.$$

Finally the full likelihood can be written as

$$\boxed{L(\phi) = \prod_{i=1}^{G} \int_{\mathbb{R}^2} \left\{ \prod_{j=1}^{K_i} \left[\int_{\mathbb{R}^2} \left(p_\phi\left(Y_{ij}, \delta_{ij} \mid M_{ij}, \nu_i, \omega_{ij}\right) \times \prod_{k=1}^{n_{ij}} p_\phi\left(M_{ijk} \mid \nu_i, \omega_{ij}\right) f(\omega_{ij}) \right) \mathrm{d}\omega_{ij} \right] \right\} f(\nu_i) \mathrm{d}\nu_i.}$$

Since $\lambda_{0,T}$ is estimated using splines, a penalized likelihood may instead be maximized:

$$l_{\text{pen}}(\phi) = l(\phi) - \kappa \int_0^\infty \frac{\mathrm{d}^2 \lambda_{0,T}(u)}{\mathrm{d}^2 u} \mathrm{d}u.$$

In practice maximization of $l_{\text{pen}}(\phi)$ is carried out using the Marquardt-Levenberg algorithm (Marquardt, 1963). The inverse of the Hessian of $l_{\text{pen}}(\phi)$ evaluated at the maximum likelihood estimator $\hat{\phi}$ can be used as an estimate of the covariance matrix of $\hat{\phi}$.

Numerical approximations

The integral over the distribution of $\omega_{ij} = (\omega_{ij0}, \dots, \omega_{ijp})$ is numerically approximated using Gauss-Hermite quadrature.

$$\begin{aligned} I_\omega &= \int_{\mathbb{R}^p} \left(p_\phi \left(Y_{ij}, \delta_{ij} \mid M_{ij}, \nu_i, \omega_{ij}\right) \times \prod_{k=1}^{n_{ij}} p_\phi \left(M_{ijk} \mid \nu_i, \omega_{ij}\right) \right) f(\omega_{ij}) \mathrm{d}\omega_{ij} \\ &\approx \sum_{l_1, \dots, l_p = 1}^{q} \Bigg(p_\phi \left(Y_{ij}, \delta_{ij} \mid M_{ij}, \nu_i, x_{l_1}, \dots, x_{l_p}\right) \times \prod_{k=1}^{n_{ij}} p_\phi \left(M_{ijk} \mid \nu_i, x_{l_1}, \dots, x_{l_p}\right) \\ &\quad \times f\left(x_{l_1}, \dots, x_{l_p}\right) w_{l_1} \dots w_{l_p} \mu\left(x_{l_1}, \dots, x_{l_p}\right) \Bigg) \end{aligned}$$

where $(x_{l_1}, \dots, x_{l_p})$ and $(w_1 \dots, w_p)$ are the nodes and weights of the Gauss-Hermite quadrature and $\mu(\cdot)$ is a normalizing function. A more accurate approximation can is achieved by using pseudo-adaptive quadrature Rizopoulos, 2012. The outer integral over the trial-level random effects distribution is carried out through Monte-Carlo integration. Let A be the (upper) cholesky decomposition of Σ_ν, that is $\Sigma_\nu = AA^\mathsf{T}$. If $X \sim \mathcal{N}(0, I_2)$, then $AX \sim \mathcal{N}(0, \Sigma_\nu)$. Thus,

$$\begin{aligned} I_\nu &= \int_{\mathbb{R}^2} \left\{ \prod_{j=1}^{n_i} \left[\int_{\mathbb{R}^2} \left(p_\phi \left(Y_{ij}, \delta_{ij} \mid M_{ij}, \nu_i, \omega_{ij}\right) \times \prod_{k=1}^{n_{ij}} p_\phi \left(M_{ijk} \mid \nu_i, \omega_{ij}\right) f(\omega_{ij}) \right) \mathrm{d}\omega_{ij} \right] \right\} f(\nu_i) \mathrm{d}\nu_i \\ &\approx \frac{1}{N} \sum_{l=1}^{N} \left\{ \prod_{j=1}^{K_i} \left[\int_{\mathbb{R}^2} \left(p_\phi \left(Y_{ij}, \delta_{ij} \mid M_{ij}, \nu_l, \omega_{ij}\right) \times \prod_{k=1}^{n_{ij}} p_\phi \left(M_{ijk} \mid \nu_l, \omega_{ij}\right) f(\omega_{ij}) \right) \mathrm{d}\omega_{ij} \right] \right\} \end{aligned}$$

where $\nu_1, \dots, \nu_n$ were generated as ν_i as $\nu_i = AX_i$ with $X_i \sim \mathcal{N}(0, I_2)$.

Numerical underflow

It may happends that if for a trial i the number of subject K_i is large or if there are many observations per individuals (n_{ij}) that the integrand $\prod_{j=1}^{K_i} \left[\int_{\mathbb{R}^2} \left(p_\phi \left(T_{ij}^*, \delta_{ij} \mid \tilde{M}_{ij}, \nu_i, \omega_{ij} \right) \times \prod_{k=1}^{n_{ij}} p_\phi \left(\tilde{M}_{ijk} \mid \nu_i, \omega_{ij} \right) f(\omega_{ij}) \right) \mathrm{d}\omega_{ij} \right]$ is so small it results in a computation underflow: it can no longer be represented by floating point numbers and is rounded to 0 by the program. This is a common issue for hierarchical likelihoods. The usual way to overcome this is to use the log function. Here this solution cannot be apploed since there the quantity in question is within an integral and $\log(\int f) \neq \int \log(f)$. One possibility is to weight each individidual contribution by a (large) constant C so that the product no longer results in an underflow. This constant must not directly depends on the values of the individual densities since it cannot longer be extrated out the integral (it will depends on

the ν_i and ω_{ij} that were're integrating over). Moreover, if this constant is fixed throughout the maximization process it may happen that this constant works well for some values of the parameter ϕ but as the iterations goes and the parameters are updated, the scale of the densities may change and C in that case might not be appropriate anymore (either too large or not enough).

Web Appendix B: Detailed simulation scheme

Let be K be the number of trials and n the total number of subjects in the dataset. The simulated model is:

$$\begin{cases} \tilde{M}_{ij}^{z}(t) = \theta_{ij}^{'} f(t) + +(\beta_{Z,M} + \nu_{M,i})z + \beta_2 z \times t + \beta_M' X_{ij}^M + \varepsilon_{ij}(t) \\ \lambda_{ij}^{zM^z}(t) = \lambda \exp\left((\beta_{Z,T} + \nu_{T,i})\, z + \beta_T' X_{ij}^T + \eta M_{ij}^z(t)\right) \end{cases} \quad (2)$$

where $\varepsilon_{ij}(t) \sim \mathcal{N}(0, \sigma^2)$ and

$$f(t) = \begin{pmatrix} 1 \\ t \end{pmatrix}, \quad \theta_{ij} = \begin{pmatrix} \beta_0 + \omega_{ij0} \\ \beta_1 + \omega_{ij1} \end{pmatrix}.$$

Parameters β_0, β_1 and β_2 are respectively the fixed intercept, slope and the effect of the treatment on the slope. The individual-level random effects ω_{ij} were generated from a bivariate gaussian distribution with mean 0 and covariance matrix

$$\begin{pmatrix} \omega_{ij0} \\ \omega_{ij1} \end{pmatrix} \sim \mathcal{N}(0, \Sigma_\omega), \quad \Sigma_\omega = \begin{pmatrix} \sigma_{\omega_1}^2 & \sigma_{\omega_{12}} \\ \sigma_{\omega_{12}} & \sigma_{\omega_2}^2 \end{pmatrix}.$$

The trial level random effects were simulated from a centered bivariate gaussian distribution with mean 0 and covariance matrix

$$\begin{pmatrix} \nu_{M,i} \\ \nu_{T,i} \end{pmatrix} \sim \mathcal{N}(0, \Sigma_\nu), \quad \Sigma_\nu = \begin{pmatrix} \sigma_{\nu_M}^2 & \sigma_{\nu_{M,T}} \\ \sigma_{\nu_{M,T}} & \sigma_{\nu_T}^2 \end{pmatrix}.$$

The different steps for simulating the datasets according to the parameters of the model are:

1. Generate $\begin{pmatrix} \nu_{M,i} \\ \nu_{T_i} \end{pmatrix}_{1 \le i \le K}$ where $\begin{pmatrix} v_{M,i} \\ v_{T,i} \end{pmatrix} \sim \mathcal{N}\left(\begin{pmatrix} 0 \\ 0 \end{pmatrix}, \Sigma = \begin{pmatrix} \sigma_{v_S}^2 & \sigma_{V_{ST}} \\ \sigma_{V_{ST}} & \sigma_{V_T}^2 \end{pmatrix}\right)$
2. For $j = 1, \ldots, n$:
   (a) Draw i uniformly from $\{1, \ldots, K\}$
   (b) Draw $\begin{pmatrix} \omega_{ij0} \\ \omega_{ij1} \end{pmatrix} \sim \mathcal{N}(0, \Sigma_\omega)$
   (c) Draw $Z_{ij} \sim \mathcal{B}(p = 0.5)$ and $X_{ij} \sim \mathcal{B}(p = 0.5)$
   (d) Generate $t_{ijk} \sim \mathcal{U}[1, 5]$ for $k = 1, \ldots, 6$ with $t_{ij0} = 0$ and $t_{ijk} \ge t_{ij(k-1)}$.
   (e) For $k = 1, \ldots, 6$:
      - Compute $M_{ij}(t_{ijk}) = \theta_{ij}^{'} f(t_{ijk}) + +(\beta_{Z,M} + \nu_{M,i}) Z_{ij} + \beta_2 Z_{ij} \times t_{ijk} + \beta_M X_{ij}$
      - Generate $\varepsilon_{ij}(t_{ijk}) \sim \mathcal{N}(0, \sigma^2)$
      - Take $\tilde{M}(t_{ijk}) = M_{ij}(t_{ijk}) + \varepsilon_{ij}(t_{ijk})$

(f) Generate T_{ij} for $j = 1, \dots, n$

(g) Generate $C_{ij} \sim \mathcal{U}[1, 6]$

(h) Retrieve the censored time $T^*_{ij} = \min(T_{ij}, C_{ij})$ and indicator $\delta_{ij} = I(T_{ij} \leq C_{ij})$

(i) Retrieve the observed longitudinal marker $\tilde{M}_{ij}(t_{ijk})$ for which $t_{ijk} \leq T^*_{ij}$.

In step 3(f), T_{ij} is generated using inverse transform sampling. More precisely, the hazard function of T_{ij} is given by

$$\lambda \exp\left(\left(\beta_{Z,T} + \nu_{T,i}\right) Z_{ij} + \beta_T' X_{ij}^T + \eta M_{ij}(t)\right) = \lambda_{ij} \exp(\eta M_{ij}(t)).$$

Moreover, the time dependency of $M_{ij}(t)$ is of the form $\alpha_{ij} t$, which $\alpha_{ij} = (\beta_1 + \omega_{ij1} + \beta_2 Z_{ij})t$. Therefore,

$$\lambda_{ij} \exp(\eta M_{ij}(t)) = \lambda'_{ij} \exp(\alpha'_{ij} t).$$

The survival function of T_{ij}, $S_{ij}(t)$, can therefore be derived as

$$\begin{aligned} S_{ij}(t) &= \mathbb{P}(T_{ij} > t) \\ &= \exp\left(-\int_0^t \lambda'_{ij} \exp(\alpha'_{ij} u) \mathrm{d}u\right) \\ &= \exp\left(-\frac{\lambda'_{ij}}{\alpha'_{ij}} \left[\exp(\alpha'_{ij} u)\right]_0^t\right) \\ &= \exp\left(-\frac{\lambda'_{ij}}{\alpha'_{ij}} \left(\exp(\alpha'_{ij} t) - 1\right)\right). \end{aligned}$$

This function can easily be inverted as:

$$S_{ij}^{-1}(q) = \frac{1}{\alpha'_{ij}} \log\left(1 - \frac{\lambda'_{ij}}{\alpha'_{ij}} \log(q)\right)$$

and therefore one can generate T_{ij} as $T_{ij} = S_{ij}(U)$ with $U \sim \mathcal{U}[0, 1]$.

Web Appendix C: Additional tables and figures

Web Appendix D: Illustration outputs

```
data(colorectal)
data(colorectalLongi)
colorectalSurv <- subset(colorectal, new.lesions == 0)

colorectalSurv$treatment<-sapply(colorectalSurv$treatment,function(t) ifelse(t=="S",1,0))
colorectalLongi$treatment<-sapply(colorectalLongi$treatment,function(t) ifelse(t=="S",1,0))
```

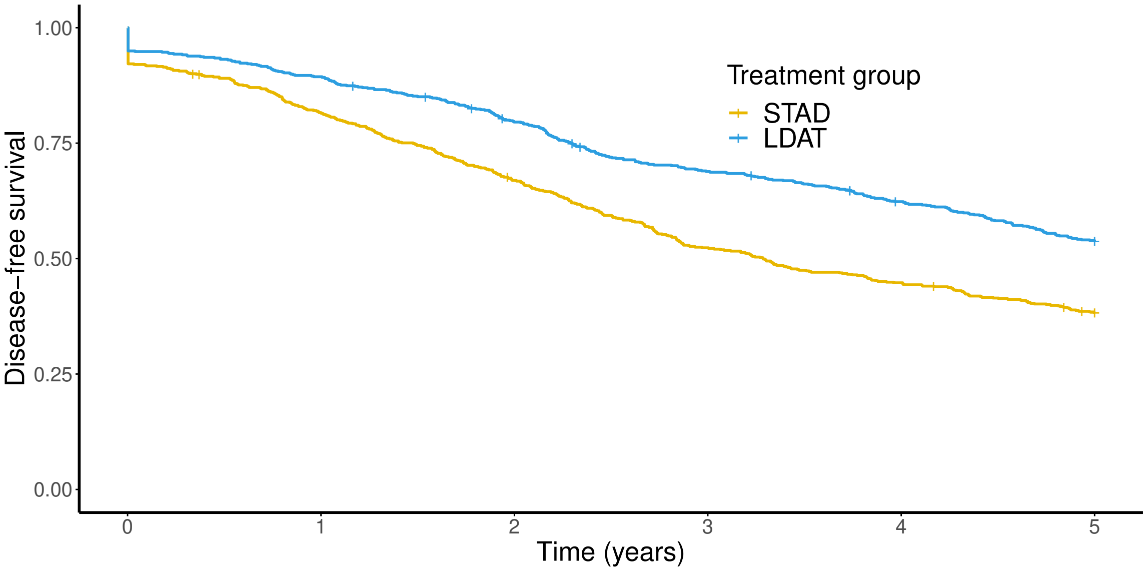

Figure 1: Disease-free survival by treatment arm

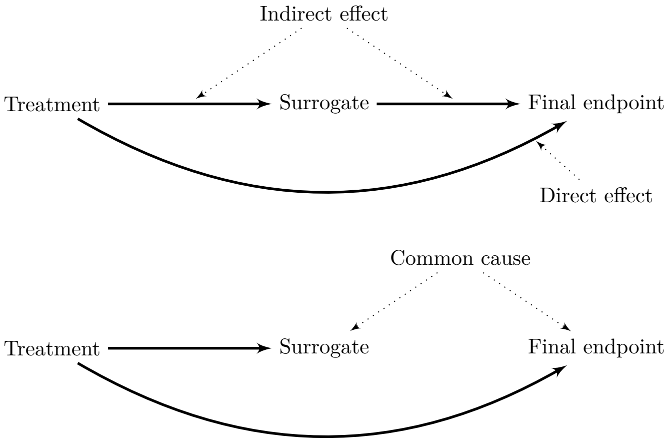

Figure 2: Illustration of how a surrogate can be validated with or without an indirect treatment effect.

```
mod.col=longiPenal(Surv(time1, state) ~ age+treatment,
                   tumor.size ~ age+year*treatment,
                   data=colorectalSurv, data.Longi = colorectalLongi, random = c("1", "year"),
                   id = "id", link = "Current-level",timevar="year",method.GH = "Pseudo-adaptive",
                   mediation = TRUE,med.trt = colorectalSurv$treatment,
                   med.center = NULL,med.nmc = 1000,n.knots = 7, kappa = 5,n.nodes = 9,
                   pte.times=c(1,1.5,2),pte.boot = F,pte.nmc = 5000,pte.nboot = 1000)

print(mod.col)
```

```
Call:
longiPenal(formula = Surv(time1, state) ~ age + treatment, formula.LongitudinalData = tumor.size ~
    age + year * treatment, data = colorectalSurv, data.Longi = colorectalLongi,
    random = c("1", "year"), id = "id", link = "Current-level",
    timevar = "year", n.knots = 7, kappa = 5, mediation = TRUE,
    med.center = NULL, med.trt = colorectalSurv$treatment, method.GH = "Pseudo-adaptive",
    n.nodes = 9, med.nmc = 1000, pte.times = c(1, 1.5, 2), pte.nmc = 5000,
    pte.boot = F, pte.nboot = 1000)


  Joint Model for Longitudinal Data and a Terminal Event
  Parameter estimates using a Penalized Likelihood on the hazard function
  Proportion of treatment effect estimated using mediation analysis

Longitudinal outcome:
----------------
                    coef SE coef (H) SE coef (HIH)         z          p
Intercept       2.882058    0.165581      0.165558 17.405682     <1e-16
age60-69 years  0.629140    0.180653      0.180644  3.482585 4.9660e-04
age>69 years    0.457796    0.144647      0.144639  3.164925 1.5512e-03
year           -0.594546    0.123914      0.123866 -4.798051 1.6022e-06
treatment       0.059103    0.215084      0.215080  0.274790 7.8348e-01
year:treatment  0.568260    0.180019      0.180004  3.156666 1.5958e-03

      chisq df global p
age 14.1014  2 0.000867

Terminal event:
-------------
                    coef exp(coef) SE coef (H) SE coef (HIH)         z          p
age60-69 years -0.190423  0.826610    0.238702      0.236851 -0.797742 4.2502e-01
age>69 years    0.060010  1.061847    0.220288      0.217090  0.272417 7.8530e-01
treatment      -0.096751  0.907782    0.200022      0.199558 -0.483703 6.2860e-01

      chisq df global p
age 1.09187  2    0.579
```

```
Components of Random-effects covariance matrix B1:

Intercept  2.182346 -0.332452
year      -0.332452  0.705785

Association parameters:
                  coef        SE       z          p
Current level 0.384876 0.0768598 5.00751 5.5139e-07

Residual standard error:  0.933346  (SE (H):  0.025974 )

Mediation analysis:
-------------



Estimated PTE, natural direct, indirect and total effect at 3 time points

  Time    PTE   Total Direct Indirect
1  1.0 3.0333 -0.0147 0.0299  -0.0447
2  1.5 2.0243 -0.0208 0.0213  -0.0422
3  2.0 1.5551 -0.0181 0.0100  -0.0281

      penalized marginal log-likelihood = -1675.92
      Convergence criteria:
      parameters = 5.65e-06 likelihood = 0.000157 gradient = 1.63e-06

      LCV = the approximate likelihood cross-validation criterion
            in the semi parametrical case     = 1.60377

      n= 150
      n repeated measurements= 906
      n events= 121
      number of iterations:  18

      Exact number of knots used:  7
      Value of the smoothing parameter:  5
```

```
Gaussian quadrature method:  Pseudo-adaptive with 9 nodes
```

References

Marquardt, D. W. (1963). An algorithm for least-squares estimation of nonlinear parameters. *Journal of the society for Industrial and Applied Mathematics* **11**, 431–441.

Rizopoulos, D. (2012). Fast fitting of joint models for longitudinal and event time data using a pseudo-adaptive Gaussian quadrature rule. *Computational Statistics & Data Analysis* **56**, 491–501.
