# Supplementary material for: Validation of a Longitudinal Marker as a Surrogate Using Mediation Analysis and Joint Modeling: Evolution of the PSA as a Surrogate of the Disease‐Free Survival
Source: Biom J. 2025 Jun 27;67(4):e70064. doi: 10.1002/bimj.70064 (PMC12205229; doi:10.1002/bimj.70064)
Supplement: Supplementary file 2 — Supporting file 2: bimj70064‐sup‐0002‐Datacode.zip. [file BIMJ-67-e70064-s001.zip › frailtypack_3.6.5.tar.gz!/frailtypack/inst/doc/Package_summary.html]

Package\_summary.knit


|  | Cox | Shared | Nested | Additive | Joint standard *(Bivariate: 1 RE + 1 TE)* | Joint cluster *(Bivariate: 1 RE + 1 TE)* | Joint general *(Bivariate: 1 RE + 1 TE)* | Joint nested *(Bivariate: 1 RE + 1 TE)* | Joint longitudinal *(Bivariate: 1 LO + 1 TE)* | Joint trivariate *(Trivariate: 1 LO + 1 RE + 1 TE)* | Joint non linear trivariate *(Trivariate: 1 LO + 1 RE + 1 TE)* | Joint Multivariate *(Trivariate: 2 RE + 1 TE)* | Joint surrogate *(Bivariate: 2 TE)* | Conditional two-part joint *(Trivariate: 2 LO 1 TE)* | Marginal two-part joint *(Trivariate: 2 LO 1 TE)* | Shared Frailty GSM | Joint Frailty GSM *(Bivariate: 1 RE + 1 TE)* |
| --- | --- | --- | --- | --- | --- | --- | --- | --- | --- | --- | --- | --- | --- | --- | --- | --- | --- |
| `Available options` |  |  |  |  |  |  |  |  |  |  |  |  |  |  |  |  |  |
| Family (PHM, AHM, POM, PROM) |  |  |  |  |  |  |  |  |  |  |  |  |  |  |  |  |  |
| Gamma distribution |  |  |  |  |  |  |  |  |  |  |  |  |  |  |  |  |  |
| Log-Normal distribution |  |  |  |  |  |  |  |  |  |  |  |  |  |  |  |  |  |
| Left-truncation |  |  |  |  |  |  |  |  |  |  |  |  |  |  |  |  |  |
| Interval Censoring |  |  |  |  |  |  |  |  |  |  |  |  |  |  |  |  |  |
| Two strata |  |  |  |  |  |  |  |  |  |  |  |  |  |  |  |  |  |
| More strata (max=6) |  |  |  |  |  |  |  |  |  |  |  |  |  |  |  |  |  |
| Time-dependant covariates |  |  |  |  |  |  |  |  |  |  |  |  |  |  |  |  |  |
| Calendar timescale |  |  |  |  |  |  |  |  |  |  |  |  |  |  |  |  |  |
| Weibull |  |  |  |  |  |  |  |  |  |  |  |  |  |  |  |  |  |
| Log-logistic, log-normal |  |  |  |  |  |  |  |  |  |  |  |  |  |  |  |  |  |
| Piecewise |  |  |  |  |  |  |  |  |  |  |  |  |  |  |  |  |  |
| `Available output` |  |  |  |  |  |  |  |  |  |  |  |  |  |  |  |  |  |
| Predicted frailties |  |  |  |  |  |  |  |  |  |  |  |  |  |  |  |  |  |
| Variances of the frailties |  |  |  |  |  |  |  |  |  |  |  |  |  |  |  |  |  |
| Martingale residuals |  |  |  |  |  |  |  |  |  |  |  |  |  |  |  |  |  |
| `Prediction methods` |  |  |  |  |  |  |  |  |  |  |  |  |  |  |  |  |  |
| Marginal prediction of a terminal event |  |  |  |  |  |  |  |  |  |  |  |  |  |  |  |  |  |
| Conditional prediction of a terminal event |  |  |  |  |  |  |  |  |  |  |  |  |  |  |  |  |  |
| Marginal prediction of a new recurrent event |  |  |  |  |  |  |  |  |  |  |  |  |  |  |  |  |  |
| Conditional prediction of a new recurrent event |  |  |  |  |  |  |  |  |  |  |  |  |  |  |  |  |  |
| `Model evaluation` |  |  |  |  |  |  |  |  |  |  |  |  |  |  |  |  |  |
| Cmeasures |  |  |  |  |  |  |  |  |  |  |  |  |  |  |  |  |  |
| Epoce |  |  |  |  |  |  |  |  |  |  |  |  |  |  |  |  |  |
| `Model structure` |  |  |  |  |  |  |  |  |  |  |  |  |  |  |  |  |  |
| STE |  |  |  |  |  |  |  |  |  |  |  |  |  |  |  |  |  |
| Prediction of treatment effects |  |  |  |  |  |  |  |  |  |  |  |  |  |  |  |  |  |
| Statistical model |  |  |  |  |  |  |  |  |  |  |  |  |  |  |  |  |  |
| Mechanistic model (ODE) |  |  |  |  |  |  |  |  |  |  |  |  |  |  |  |  |  |
| Mediation analysis |  |  |  |  |  |  |  |  |  |  |  |  |  |  |  |  |  |

Table 1: Package characteristics. Blue cross is for option available
for a given type of model in the package on CRAN, orange cross is for
option included in the package but not on CRAN yet. Empty cells mean
that an option is not available for a given type of model (either not
coded yet or simply not applicable). RE = Recurrent Event; TE = Terminal
Event; LO = Longitudinal Outcome; STE = Surrogate threshold effect; ODE
= Ordinary Differential Equation; GSM = Generalized Survival Model; PHM
= Proportional Hazards Model; AHM = Additive Hazards Model; POM =
Proportional Odds Model; PROM = Probit Model.

#### Reference

Agnieszka Krol, Audrey Mauguen, Yassin Mazroui, Alexandre Laurent,
Stefan Michiels, Virginie Rondeau (2017), Tutorial in Joint
Modeling and Prediction: A Statistical Software for Correlated
Longitudinal Outcomes, Recurrent Events and a Terminal Event.
*Journal of Statistical Software*, **81**.

Casimir Ledoux Sofeu and Virginie Rondeau (2020). [How to use
frailtypack for validating failure-time surrogate endpoints using
individual patient data from meta-analyses of randomized controlled
trials.] (https://doi.org/10.1371/journal.pone.0228098) *PLOS
ONE*, **15**, 1-25.

#### Shiny

A shiny application of frailtypack is available, allowing
modelisation and prediction of several models presents in frailtypack,
at https://frailtypack-pkg.shinyapps.io/shiny\_frailtypack/
This application can be run in a local mode in R thanks to the function
runShiny().

##### Note

If you want to take in charge frailtypack as a project, but work on a
macOSX with a version of R >= 3.6, a problem must happen between
openmp and clang. clang does not seem to support option ‘-fopenmp’. To
avoid this, you should edit your personal Makevars (~/.R/Makevars),
admitting you use gfortran, by relocating CC=clang by CC=gfortran
